# Phylogeography and diversification history of the day-gecko genus *Phelsuma* in the Seychelles islands

**DOI:** 10.1186/1471-2148-13-3

**Published:** 2013-01-05

**Authors:** Sara Rocha, David Posada, D James Harris

**Affiliations:** 1 CIBIO-UP, Centro de Investigação em Biodiversidade e Recursos Genéticos, Campus Agrário de Vairão, Vairão 4485-661, Portugal; 2 Departamento de Biologia, Faculdade de Ciências, Rua do Campo Alegre, Porto FC4 4169-007, Portugal; 3 Departamento de Bioquímica, Genética e Inmunología, Facultad de Biología, Universidad de Vigo, Vigo 36310, Spain

**Keywords:** *Phelsuma*, Seychelles, Phylogeography, Species-trees, Diversification, Morphological evolution, Character displacement, Biogeography

## Abstract

**Background:**

Lying in a shallow continental shelf cyclically affected by oscillating sea levels since the Miocene, the Seychelles islands are particularly interesting for evolutionary studies. Recent molecular studies are generating an emerging picture of the origin of its biota, yet very little is known regarding their phylogeographic structure or on the factors promoting diversification within the archipelago. Here we aimed to obtain a detailed depiction of the genetic structure and evolution of one of the most widespread vertebrate groups in the archipelago: the day-geckos of the genus *Phelsuma*. In parallel, we aimed to infer divergence times between species and subspecies, testing a long-standing hypothesis that argues for different time since sympatry between species as the cause of their different morphological differentiation across the archipelago.

**Results:**

Molecular data corroborated the existence of two main lineages, corresponding to the two currently recognized species. Divergences between species likely date back to the Mio-Pliocene, while more recent, Pleistocenic, divergences are suggested within each species. Populations from outer islands share mtDNA haplotypes with inner island populations, suggesting very recent dispersals (or introductions). We found no evidence of current gene flow between species, but results pointed to the possibility of gene flow between (now allopatric) subspecies. Time estimates suggest a synchronous divergence within each species (between island groups).

**Conclusions:**

The geographic patterns of genetic variation agree with previous taxonomic subdivisions within each species and the origin of outer islands populations is clearly tracked. The similar intraspecific divergence time estimates obtained suggest that the differential body-size differentiation between species within each group of islands may be driven by factors other than character displacement proportional to time since sympatry, as previously suggested. These factors could include different habitats/resources available within each island group, niche differentiation and/or character displacement. We also bring again into consideration the hypothesis of body size being influenced by the distribution of native vegetation and social systems within this group, although it remains to be tested. Our results highlight not only the necessity of clarifying the role of ecology and interspecific interactions in this group’s morphological diversification and community assemblage, but also the importance of co-evolutionary mechanisms and their importance for appropriate conservation of island biodiversity. Further, we provide a detailed description of the phylogeographic structure of these taxa across these islands, which still remain poorly characterized in this respect.

## Background

**Figure 1:**
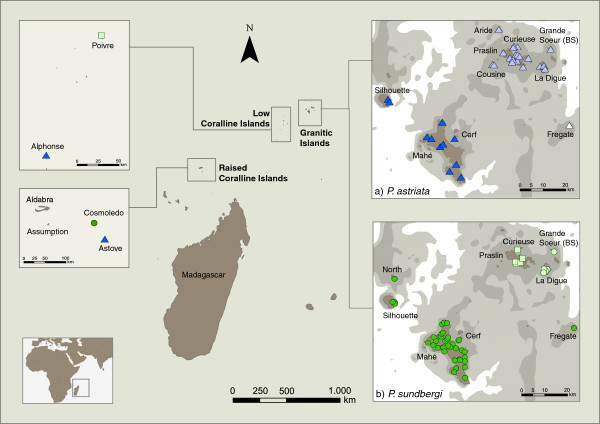
**Sampling sites for *Phelsuma *species along the granitic and coralline Seychelles islands.** Species and subspecies are coded using different symbols: dark blue triangles - *P. astriata astriata*; light blue triangles - *P. astriata semicarinata*; white triangle - *P. astriata* (Fregate population); dark green circles - *P. sundbergi longinsulae*; light green squares - *P. sundbergi sundbergi*; light green circles - *P. sundbergi ladiguensis*. Within the granitic islands (insets **a** and **b**), different shadings show areas that would be emerged at −30 m (dark grey) and −50 m (light grey) below present sea-level stands. Low-stands below 60 m would allow contact between all islands in this granitic group. Current data on Indian Ocean sea level changes support low-stands of up to 145 ± 5 m below present sea level (bpsl) at six episodes in the last 0.5 Myr, some of which persisted for up to 50,000 years at a time see [6] and geological records going back further suggest low-stands of 80–120 m bpsl at 0.64, 0.88, 1.04, 1.26 and 1.54 Mya, and eleven additional low-stand episodes of 50 m or more bpsl during the last 5 Myr [18]. Detailed locations and accession numbers are given in Additional file 1.

The Western Indian Ocean islands of Madagascar, Comoros, the Mascarenes, and the Seychelles are one of the 34 “hotspots” defined by Conservation International [1]. While there is an emerging picture from molecular data about the origin of their biota (see [2-7] for recent reviews), diversification mechanisms within each archipelago and island are less understood. That is particularly true for the Seychelles, where only a handful of studies have explored the genetic structure of different species within the archipelago [8-13]. The separation of the Seychelles and the Indian subcontinent is estimated to have been completed 65 Mya and the Seychelles include about 155 islands that can be divided in 3 groups: granitic, low and raised coralline islands (Figure 1). The granitics are a group of about 40, clustered together on the undersea shelf of granite, that is the Seychelles bank. These comprise the islands of North, Silhouette, Mahé, Fregate, Praslin, La Digue, Curieuse and several smaller ones encircling them. The low coralline islands are very recent, probably less than 6,000 years ago, and formed from marine sediments cemented with guano deposits [14]. The raised coralline islands (Aldabra, Assumption, Astove and Cosmoledo) are also oceanic, formed by reef-building corals acting on submerged volcanic seamounts that may have formed some 20 million years ago (Mya) [15] and were submerged and re-emerged several times since their formation.

Sea-level oscillations occurred throughout the Pleistocene and back through the Miocene, with low stands of up to 145 m below present sea-level (bpsl) [16-18]. All the islands in the plateau would have been united with sea-levels of roughly 60 m bpsl, but even less pronounced minima greatly increased the size of each island and their connectivity (Figure 1a, b). It is probable that these cycles of allopatry and contact between islands shaped distribution patterns, organismal divergence and diversification within the archipelago. Nevertheless, intra-archipelago genetic structure of Seychellois taxa is poorly known, as well as the relative influence of isolating vs. connecting periods on species diversification. As in other archipelagos cyclically isolated and connected, cryptic diversity might be higher than currently appreciated [19,20].

Historically, Mahé and surrounding islands, plus Silhouette and North have been considered to form a biogeographic unit separated from the Praslin-La Digue (and surrounding islands) group. Fregate, located roughly in the middle of the two groups of islands, is generally regarded as an isolated or intermediate unit [8,21,22]. Distribution data for insects [21], molluscs [23], and a few studies of intraspecific variation within amphibians [24] and reptiles [8,25] are consistent with this pattern. However, this does not seem to stand for some other groups [9,26]. Most of the few existing studies on intraspecific genetic variation suggest deep and cryptic divergences among the granitic Seychelles [12,13,27,28], highlighting the need for thorough sampling and phylogeographic analyses across these islands. At the same time, it becomes important to understand also the relative influence of historical and ecological factors in the generation and maintenance of such diversity. Day-geckos of the genus *Phelsuma* are ideal in this respect, as its two Seychellois species are widespread across most of the islands, with both sympatric and allopatric populations. *Phelsuma* mostly diversified in Madagascar, from where they colonized the surrounding archipelagos and often speciated (reviewed in [28]). Within each island clade, *Phelsuma* species diverged ecologically and morphologically, evolving a wide variety of colour patterns and sizes and occupying a broad range of arboreal habitats (see [29] and references therein). However the relative contribution of geographic isolation, local adaptation and interspecific interactions, including hybridization, in the generation of these species assemblages is rather unclear.

In the granitic Seychelles, *Phelsuma* is represented by a small endemic clade [8,30]. The classification of the Seychelles “forms” of *Phelsuma* has been a persistent problem due to the considerable variation in colour pattern and size across islands. Cheke [31] and Gardner [25,32,33] assessed the systematics of the Seychelles forms and currently two endemic species are recognized with an unclear number of subspecies. Within *P. sundbergi*, up to six subspecies have been recognized: *P. sundbergi longinsulae*, inhabiting Mahé (and associated islands) plus Fregate; *P. (sundbergi) longinsulae rubra* and *umbrae*, respectively from North and Silhouette sensu [34] reflecting previous consideration of *P. longinsulae* as a separate species; *P. s. sundbergi,* from Praslin, Curieuse, Aride and surrounding islands, and *P. s. ladiguensis* from La Digue, Mariane, Grande and Petite Soeur and surrounding islands (Figure 1). Gardner [25,33] examined variation within these species based on body size, scalation and colour pattern variables and found support for only three of the groups, *P. s. longinsulae*, *P. s. sundbergi* and *P. s. ladiguensis* (the latter two being clearly more similar), but not for the distinction of the North and Silhouette forms, *rubra* and *umbrae*. These were synonymised with *P. sundbergi longinsulae*, as was also *P. (sundbergi) longinsulae menaiensis* sensu [35], a putative subspecies from the coralline islands of Cosmoledo described in some detail by Cheke [31]. Within *P. astriata* two allopatric subspecies are recognized; *P. a. astriata* and *P. a. semicarinata*, from the southern and northern group of islands respectively (Figure 1), with populations from Fregate described as “intermediate forms” and hypothesized to be hybrids between the two subspecies [31]. Individuals from Astove (raised coralline island) were considered by Cheke [31] to be closely related to the nominate *astriata* on Mahé.

The degree of morphological differentiation between and within species and subspecies varies geographically. Notably, body size variation shows no clear geographic pattern within *P. astriata*, while clearly increases from south to north within *P. sundbergi*; with *P. s. sundbergi* and *P. s. ladiguensis* individuals being significantly larger than *P. s. longinsulae*[8,32]. Radtkey [8] proposed allopatric speciation followed by asynchronous dispersals across island groups plus resource competition generating character displacement as the drivers of speciation and body size evolution within this group. More specifically, he proposed that after the invasion of the Seychelles archipelago by a single species of *Phelsuma*, sea-level changes led first to allopatric speciation, with the ancestor of *P. sundbergi* and *P. astriata* differentiating in the southern and northern groups of islands, respectively, and then to the asynchronous dispersal of these species across island groups. The differences in the relative timing of the dispersal between island groups (with the ancestor of *P. s. sundbergi* and *P. s. ladiguensis* colonizing the northern group of islands much earlier than the ancestor of *P. astriata astriata* colonized the southern group) would have resulted in *P. sundbergi* evolving an intermediate body size in the group of islands associated with Mahé (southern) and a large body size in the group of islands associated with Praslin and La Digue (northern), due to character displacement, and given its longer period of sympatry with *P. astriata* in the northern islands. Nevertheless, this is not the only possible explanation for the different degrees of body size disparity. Body size distributions may be governed instead by different ecological variables and interactions happening within each island group [32,36]. For example, competition and selective pressures for body size within each island or island group may be distinct and lead to distinct patterns of morphological differentiation between species within island group, independently of the time since sympatry. In fact, under character displacement, phenotypic divergence accelerates in sympatry [37] but needs not be correlated with time since sympatry [38,39].

Gardner [32] proposed instead that body size in Seychellois *Phelsuma* has probably been influenced by the distribution of vegetation and social systems. The use of defensible pollen and nectar food supplies, especially the male inflorescences of *Lodoicea maldivica*, native to (and only found in) the northern islands of Praslin and Curieuse, may have resulted in the evolution of the larger body size of *P. sundbergi* in the northern islands while hardwood forests, shrub and seabird islands vegetation may have led to smaller sizes in the south. The competitive relationships between the species could then be influenced by their body size differences and help to support the selective pressures within the system. Sympatric species within each island may be partitioning their habitat differently or diversifying along different axis eg. [40-43], resulting in the observed pattern, without the need to invoke asynchronous sympatry as its cause. In the Mascarenes for example, sympatric species of *Phelsuma* often differ across several resource axes at the same time [44]. If the time since contact between the different species in the Seychelles is similar, ecological factors shall play a much more important role in morphological diversification of Seychellois *Phelsuma* than currently appreciated. It is also not known if these species hybridize on any island group.

Our objectives here were first to assess the intraspecific genetic variation within species and its geographical distribution, inferring possible colonization routes and the geographic origins of outer island populations. Second, using multilocus data, to explore the possibility of gene flow across species and subspecies and infer the underlying population’s history. Ultimately, we aimed to test the asynchronous divergence within each species, simultaneously providing a time scale for the diversification of this clade. Such a phylogeographic approach will help us to better understand the relative influence of oscillating sea-levels in shaping diversification across these islands, and also should provide a valuable framework upon which the ecological determinants of diversification within this group can be further explored.

## Results

### Mitochondrial DNA variability and genetic structure

**Figure 2:**
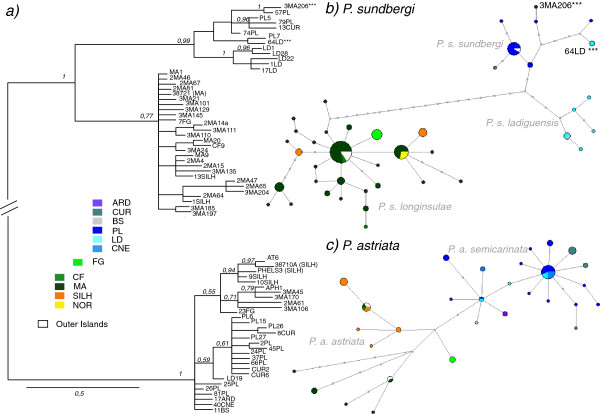
**a) MtDNA haplotype phylogeny.** Midpoint-rooted Bayesian 50% majority-rule consensus tree with posterior probabilities above 0.5 indicated above branches. The scale bar refers to branch lengths (expected changes per site). Median-Joining networks for *P. sundbergi* (**b**) and *P. astriata* (**c**). Islands are colour-coded and circle size is proportional to the number of individuals. Full grey dots along branches represent missing or unsampled haplotypes. Islands labelling is as follows: Aride (ARD), Curieuse (CUR), Grande Soeur (BS), Praslin (PL), La Digue (LD), Cousine (CNE), Fregate (FG), Cerf (CF), Mahé (MA), Silhouette (SILH) and North (NOR). Highlighted haplotypes (***) correspond to the individuals from Mahé and La Digue found to cluster within *P. sundbergi sundbergi* (from Curieuse and Praslin) (see text). The outer islands of Cosmoledo, Astove (AT), Poivre and Alphonse (APH) are identically coloured in white (see Results for detailed information).

The two species, *P. sundbergi* and *P. astriata* correspond to two divergent mtDNA clades (Figure 2) (Nei’s *Da* = 11%). Intraspecifically, *Phelsuma sundbergi longinsulae* clearly differentiates from *P. s. sundbergi* and *P. s. ladiguensis* (*Da* = 2.5%) (Figure 2b). Within *P. s. longinsulae*, the North and Silhouette populations share or carry haplotypes very closely related to Mahé. Silhouette haplotypes are paraphyletic relative to Mahé, and individuals from North exhibit a single haplotype, shared with individuals from Mahé (Figure 2b). Furthermore, all individuals from Fregate carry a unique haplotype, which is just one nucleotide substitution away from the most abundant haplotype from Mahé. The haplotype of one *P. sundbergi* individual from Mahé (3MA206) clusters with *P. sundbergi* haplotypes from Praslin. We provisionally interpreted this as a possibly anthropogenic related pattern (but see Discussion) and excluded this individual for the inference of divergence times and gene flow. Concerning the *P. sundbergi* populations from the northern group of Praslin, La Digue and associated islands, i.e., *P. sundbergi sundbergi* and *P. s. ladiguensis*, they form two slightly divergent mtDNA clades, in agreement with their morphological variation: Praslin and Curieuse populations in one clade and La Digue and Grande Soeur populations in the other clade (Figure 2b). Yet, two individuals from La Digue exhibit haplotypes that cluster within those from Praslin.

Within *P. astriata* the non-overlapping geographic distribution of mitochondrial lineages agrees also with the currently recognized subspecies (Figure 2c). The divergence between these lineages is not large, with just three substitutions separating haplotypes from *P. a. astriata* and *P. a. semicarinata,* and with two and three substitutions separating both these subspecies from individuals from Fregate, where the three individuals sampled exhibit the same mtDNA haplotype.

All the outer island populations share mtDNA haplotypes with the granitic islands populations (Figures 2b and 2c, in white colour). The Cosmoledo populations of *P. sundbergi longinsulae* exhibit a single haplotype, the central one in the phylogenetic network and the most abundant in Mahé. Similarly, *P. astriata astriata* haplotypes from Alphonse are also shared with individuals from Mahé, while the Astove population of *P. astriata astriata* shares its (again single) haplotype with Mahé, Cerf and Silhouette individuals. Finally, individuals of *P. sundbergi sundbergi* from Poivre exhibit a single and same haplotype as most of the individuals from Praslin.

### Nuclear DNA genealogies

**Figure 3:**
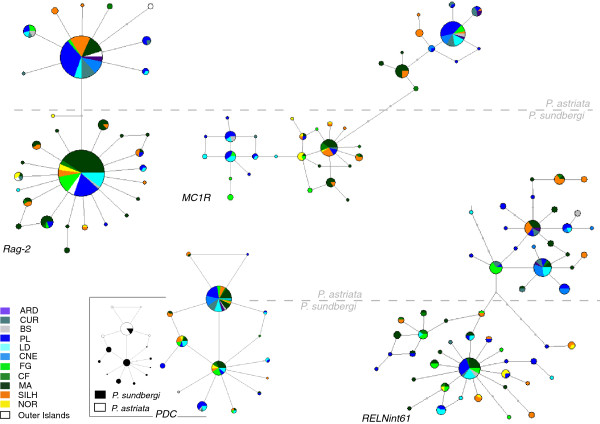
**MJ networks representing nuclear DNA variation.** Islands are colour-coded and circle size is proportional to the number of individuals. Labelling is the same as in Figure 2. Full grey dots along branches represent missing or unsampled haplotypes. Clades above and below the dashed horizontal line correspond to *P. astriata* and *P. sundbergi*, respectively (except for *PDC* where haplotype sharing is observed – inset network). The gene to which each network corresponds is identified on its inferior left corner.

The nuclear variation is almost completely sorted between species (Figure 3). *P. astriata* and *P. sundbergi* share only a single haplotype for one of the genes (*PDC*), which is central in the phylogenetic network and the most abundant in *P. astriata*. Overall, divergence between species is not high and the conspecific subspecies in all cases share a high number of haplotypes. The only nuclear gene where intraspecific structure is noticeable is *MC1R*, with two subgroups within each species, with a strong geographic component.

### Genetic variability and neutrality tests

Considerable variation was observed in the levels of polymorphism across loci (Table 1), with *Cytb* and *RELNint61* being the polymorphic ones. The variability levels within each subspecies were not remarkably different, although they were slightly higher within *P. sundbergi longinsulae*. Tajima’s D and Fu’s Fs were usually negative, while R_2_ values were usually small, suggesting demographic growth across all groups, although only in a few instances (*P. sundbergi longinsulae* for the mitochondrial and *Rag-2* markers and *P. astriata semicarinata* for *Cytb*) were values significant for all statistics.

**Table 1:** **Summary diversity statistics, tests of neutrality and indexes of populations growth for all subspecies of *P. sundbergi *and *P. astriata***

**Subspecies**	**Locus**	**Lenght**	**N**	**Polymorphism**					**Neutrality**	**Population growth**	
				**H**	**S**	**Hd**	**π**	**θw**	**D**	**F**_**S**_	**R**_**2**_
*P. sundbergi longinsulae*	*Cytb*	761	93	28	30	0.875	0.00362	0.00772	−1.62892*	−17.813*	0.0445*
	*MC1R*	617	56	13	10	0.758	0.00261	0.00358	−0.76192	−5.943*	0.0759
	*RELNint61*	877	52	19	21	0.903	0.00401	0.00587	−1.02213	−6.903*	0.0798
	*Rag-2*	796	136	17	15	0.554	0.00089	0.00345	−1.97263*	−1.97263*	0.0261*
	*PDC*	360	56	7	6	0.710	0.00255	0.00363	−0.74314	−2.145	0.0772
*P. sundbergi sundbergi*	*Cytb*	761	17	6	12	0.588	0.00234	0.00466	−1.87150*	−0.889	0.0983 *
	*MC1R*	617	22	9	7	0.853	0.00391	0.00312	0.81826	−2.265	0.1645
	*RELNint61*	877	22	12	19	0.835	0.00364	0.00596	−1.44378	−4.329*	0.0839
	*Rag-2*	796	34	6	5	0.371	0.00051	0.00154	−1.76914*	−4.537*	0.0611
	*PDC*	360	22	7	6	0.762	0.00422	0.00457	−0.23808	−1.875	0.1186
*P. sundbergi ladiguensis*	*Cytb*	761	7	5	6	0.857	0.00300	0.00322	−0.33869	−1.262	0.1543
	*MC1R*	617	10	5	4	0.844	0.00223	0.00229	−0.09820	−1.468	0.1652
	*RELNint61*	877	10	6	8	0.758	0.00211	0.00303	−1.20688	−1.540	0.1351
	*Rag-2*	796	14	2	1	0.143	0.00018	0.00040	−1.15524	−0.595	0.2575
	*PDC*	360	14	7	6	0.890	0.00394	0.00524	−0.89259	−3.209*	0.1071*
*P. astriata astriata*	*Cyt b*	761	20	10	18	0.895	0.00541	0.00667	−0.71027	−1.539	0.1044
	*MC1R*	617	30	8	8	0.660	0.00279	0.00327	−0.44757	−1.813	0.1059
	*RELNint61*	877	30	11	10	0.867	0.00356	0.00289	0,.3543	−2.134	0.1555
	*Rag-2*	796	40	3	2	0.188	0.00024	0.00059	−1.11593	−1.548	0.0858
	*PDC*	360	30	3	2	0.439	0.00132	0.00140	−0.12690	−0.044	0.1184
*P. astriata semicarinata*	*Cyt b*	761	46	19	20	0.865	0.00289	0.00598	−1.67207*	−12.687**	0.0499*
	*MC1R*	617	64	6	4	0.445	0.00084	0.00137	−0.82812	−2.856*	0.0651
	*RELNint61*	877	56	10	11	0.728	0.00307	0.00277	0.30059	−0.596	0.1193
	*Rag-2*	796	92	4	3	0.184	0.00024	0.00074	−1.23597	−2.748	0.0445
	*PDC*	360	58	3	2	0.101	0.00028	0.00120	−1.31498*	−2.516*	0.0673

^a^ N, number of individuals (mtDNA)/chromossomes (nucDNA). Polymorphism indexes: H, number of haplotypes detected; S, number of segregating sites; Hd, haplotype diversity; π, nucleotide diversity; θw, population mutation parameter [62]. Neutrality test: D, Tajima’s D. Population growth tests: F_S_, Fu’s F_S_; R_2_, Ramos-Onsins and Rozas’ R_2_.* *p* < 0.05; ** *p* < 0.01.

^b^ Statistics were not computed for *P. astriata* population from Fregate as only three individuals were sampled. Populations from outer islands and hypothetically introduced individuals (3, see above) were also excluded of calculations.

### Time and gene flow estimates under the isolation-with-migration model

**Figure 4:**
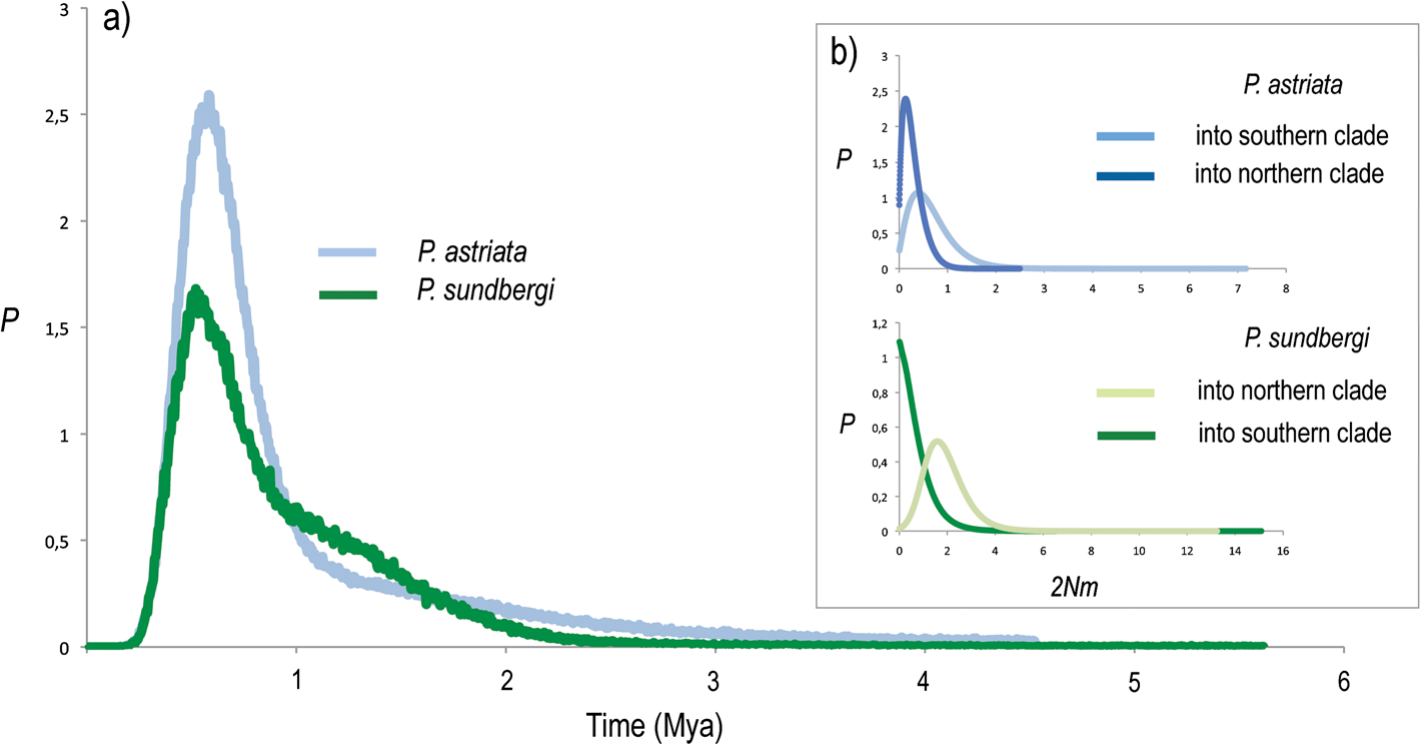
**a) Probability distribution for the splitting time between northern and southern clades of *P.  astriata *(light blue) and *P. sundbergi *(dark green); b) number of migrant gene copies per generation (2 *Nm*) between those same clades within *P. astriata *(blue, upper) and *P. sundbergi *(green, down).**

Using the IM model we obtained consistent estimates of demographic parameters across all runs (Table 2), with high effective sample size (ESS) values, convergence and a good sampling of the parameter space. Assuming a mean rate substitution of 1% per lineage per million years (Myr) at this marker, the estimated time since divergence between the two species was 6.39 Myr, with a 95% high posterior density (HPD) interval of 4.264 – 8.397. No gene flow was detected between species and the ancestral effective population size estimate was very low (Table 2, bottom row). Estimates of the splitting times between the southern and northern clades of both species were fairly coincident (~500,000 years old) as so were their posterior density intervals (Table 2 and Figure 4a), which were, nevertheless, quite large. The effective population size estimates for *P. sundbergi* doubled those for *P. astriata*, irrespectively of the island group. Accordingly, the estimated effective ancestral population sizes were also higher in *P. sundbergi*. Even having excluded the few individuals presumed to be the result of anthropogenic introductions (as mentioned above), significant migration rates were still recovered within both species, especially for *P. sundbergi* (Table 2 and Figure 4b). Neither recombination nor selection was detected at any locus.

**Table 2:** Isolation-with-migration estimates

**Parameter estimates**				
Group/Species	Ancestral Effective Population Size *Ne* [95%HPD]	Effective Population Size *Ne* [95%HPD]	Effective Number of Migrants *into the population 2 Nm* [95%HPD]	Time Since Divergence *T* [95%HPD]
	Absolute (millions of individuals)	Absolute (millions of individuals)	Absolute (migrant gene copies per generation)	Absolute (in million years)
*P. s. sundbergi + P. s. ladiguesis* (northern)	0.600 [0.104-2.452]	1.826 [1.046-3.148]	1.584 [0.3513 – 3.59]**	0.536 [0.266-1.901]
*P. sundbergi longisulae* (southern)		3.584 [2.228-5.598]	0.0075 [0 – 1.995]	
*P. astriata astriata* (northern)	0.296 [0.0014 – 0.868]	0.764 [0.424-1.280]	0.1311 [0 – 0.692]	0.580 [0.251-2.876]
*P. astriata semicarinata* (southern)		0.752 [0.390-1.436]	0.3910 [0 – 1.503] *	
*P. sundbergi*	0.002 [0 – 2.408]	2.880 [2.143 – 3.737]	0.0006 [0 – 0.1763]	6.39 [4.264 – 8.297]
*P. astriata*		0.765 [0.564 – 1.035]	0.0003 [0 – 0.08]	

Estimates and 95% highest posterior densities (HPD) of population sizes (θ), effective number of migrants (*2 Nm*) and time since divergence (*T*) between groups, as inferred with IMa2 (three different comparisons). Values for migration that are significantly different from zero [LLR test: * p-value <0.05; ** p-value <0.01, 82] are indicated with asterisk. A substitution rate of 1% per site per million years and a generation time of one year were assumed to convert the estimates into absolute quantities.

### Seychelles’ *Phelsuma* species tree

**Figure 5:**
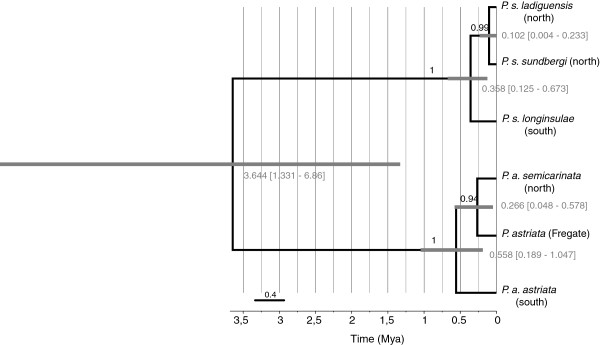
**“Species” tree of the *Phelsuma *day-geckos endemic from the Seychelles.** The tree shown corresponds to the maximum clade credibility tree with mean estimates used for node heights and it is based on the multispecies coalescent analysis of the five molecular markers and assuming a rate of evolution of 0.01 (SD = 0.0027) substitutions per lineage per My for Cytb. Clade posterior probability is indicated above each branch. Node bars correspond to the 95% high posterior density intervals for node height (age), which are given below/in front of each node. Horizontal axis corresponds to time in million years before present (Mya).

*BEAST analyses recovered consistent results across replicates, with high ESS values for all parameters of interest. Estimates without data recover completely different values and distributions, showing that the data, and not the prior, are primarily responsible for the posterior distributions. The maximum clade credibility species tree (Figure 5) was in general agreement with the inferences made under the IM model in respect to the ages of the branching events. The initial split between the two species (*P. sundbergi* and *P. astriata*) was dated to circa 3.6 Myr, although the 95% HPD age intervals were very wide (1.33 – 6.86). These estimates are slightly more recent than those obtained under the IM model (Table 2), but the HPD intervals considerably overlap. Estimates of 95% HPD intervals for the north–south split within both species again clearly overlap. The population of *P. astriata* from Fregate (included here though excluded from the IMa analysis so data could fit a two populations model) was recovered as sister taxa to *P. astriata semicarinata*, with moderate support.

## Discussion

### Species divergence and intraspecific genetic structure

Here we analyzed in detail for the first time the genetic structure of the *Phelsuma* day-geckos in the Seychelles based on a comprehensive sampling across the archipelago. Molecular data from widespread populations across these islands corroborate the two recognized species as two single main lineages and place their divergence in the late Miocene or early Pliocene. That is earlier than the Pliocene/Pleistocene divergence estimates within the similarly distributed Seychellois crab genus *Seychellum*[12] but in the same range as the north–south split within the co-distributed Seychellois gecko species *Urocotyledon inexpectata*[13].

Very recently, Carranza et al. [45] obtained rate estimates for part of the same *Cytb* fragment used in this study that are higher that the one we used (approximately the double and up to three times higher, depending on the group of taxa, although with high standard deviations). Pending further confirmation of these rate estimates, we preferred to keep the use here of a more conservative (and widespread) average rate of 1% substitutions per lineage per Myr. Still, we note the need of taking these absolute age estimates with caution. We note also that using a faster rate would lead us to more recent estimates of species and subspecies diversification (although yet early Pliocenic and Pleistocenic, respectively) but that it would not change the relative timing between these events. Importantly, it would not change the inference of a simultaneous split within each species.

We did not find evidences of past or current gene flow between the two species. While the application of the IM model is debatable given the high levels of population structure observed, Strasburg and Rieseberg [46] showed that most parameter estimates in IMa are fairly robust to population structure over a wide range of parameter values, down to *Nm* = 0.1 among conspecific populations. It is nevertheless possible that estimates of population sizes and divergence times are slightly biased upwards in these cases [46].

Within each species, our data are congruent with previous multivariate morphometric studies from Gardner [25], with Cheke’s [31] taxonomic classification and with previous inferences from Radtkey [8]: three lineages within *P. sundbergi* and two lineages within *P. astriata*. The limited number of individuals of *P. astriata* sampled from Fregate, as well as the little intraspecific differentiation found between the northern and southern islands did not allow us, though, to infer whether the individuals from Fregate are in fact the result of hybridization between distinct clades (as previously proposed) or are just a distinct, equidistant, clade.

Due to the reciprocal monophyly of the main clades within each species at the mtDNA level and the low variation and lack of sorting at the nuclear markers, it seems too speculative to depict historical colonization sequences and routes within the granitic Seychelles. Several scenarios could fit the observed patterns. Moreover, extinction and demographic changes may have affected populations differently, further obscuring any historical signal to be inferred. As argued by Gardner [32], current diversity could be explained by the sectioning of pre-existing clinal variation due to rising sea levels. It seems likely therefore that divergence between and within each species in the granitic islands was mostly driven by vicariance through cycles of allopatry between populations, rather than through stepping-stone colonization events. However, if isolation was due solely to oscillating sea-levels we would expect that the main split occurred between Silhouette and North versus remaining islands, such as in [12] (see Figure 1). It seems though, that distance was also an important factor for these species, and they were likely able to perform short overwater dispersals more often than long ones, leading to the main subdivision being between northern (Praslin-group) and southern (Mahé group, including Silhouette and North) islands.

Within *P. sundbergi*, some observations point to the possibility of (past or ongoing) gene flow between subspecies (island groups): one individual collected in Mahé as *P. s. longinsulae* exhibited in fact an haplotype clustering within *P. s. sundbergi* (from Curieuse and Praslin), and the same happened with two individuals collected in La Digue that grouped within *P. s. sundbergi*. Given that we did not collect morphological measurements or photos that would allow us to reconfirm the identification of these specimens, and given previous records of occasional dispersals that do not seem to form viable populations [31] we provisionally considered these individuals as possible cases of anthropogenic or very recent dispersals, and eliminated them from further analyses when appropriate. Nevertheless, even without considering these individuals, the IMa analyses returned positive estimates of gene flow within *P. sundbergi* (specifically, into the northern clade of *P. s. sundbergi* and *P. s. ladiguesis*), suggesting that the possibility of gene flow should be investigated in more detail. Also between *P. astriata* there were signs of possible gene flow, albeit weaker. Given the estimated Pleistocenic divergence between these subspecific clades, and low sea levels assumed during this period [6,18], which could have facilitated contact between island groups, it is reasonable to expect that gene flow may have occurred. Pending further studies shedding light on gene flow estimates and possible ecological differences across island groups, we consider the current taxonomy of this group appropriate, with only two species, *P. sundbergi and P. astriata*, that include three and two subspecies (or lineages; we are not necessarily advocates of the subspecies rank) respectively. *Phelsuma astriata* population from Fregate remains unassigned as *P. astriata ssp*.

The phylogeographic pattern of *P. sundbergi* (and *P. astriata* to a certain extent) mirrors the patterns found within *U. inexpectata*, a gecko species co-distributed with *Phelsuma* across the granitic Seychelles [13] and may thus reflect a more general biogeographic pattern. Many similarities can also be found with a co-distributed endemic freshwater crab species, *Seychellum allaudi*[12] with the difference that in this case Silhouette populations split first from the remaining populations, which seems logical due to their lower dispersal capacity.

### The origins of the outer island populations

Cheke [31] discussed the status of the populations from Cosmoledo and Astove, keeping their designation as separate subspecies (*P. longinsulae menaiensis* and *P. astriata astovei*) based on slight colour variations. Gardner [25,33] found no morphological differentiation between *P. sundbergi* populations from the coralline and granitic islands and argued for their probable recent colonization or introductions by man (in the coralline islands). Accordingly, the fact that all individuals analysed from each of these populations possess the same mtDNA haplotype, shared with populations from the granitic islands, supports their very recent origin, either by natural dispersion or as a result of human-driven introductions. Specifically, based on mtDNA variation, the population from Cosmoledo could be assigned to *P. sundbergi longinsulae*, Poivre to *P. sundbergi sundbergi*, and the populations from Astove and Alphonse to *P. astriata astriata*.

### The timing and mechanisms driving diversification of *Phelsuma* in the Seychelles

Perhaps the most unexpected result of this study was the estimate of simultaneous divergence of the northern and southern lineages within both species. In the biogeographic scenario previously proposed by Radtkey [8], the asynchronous divergence between these clades within each species would be responsible for the different degrees of morphological differentiation observed between the two species within each island group (the degree of character displacement being proportional to time since contact between the different species). Our results contradict this hypothesis, implying that other factors should be responsible for the morphological differentiation between species within each island group. The biogeographic scenario proposed by Radtkey [8] was largely based on his estimate of the mtDNA phylogeny, and on the different pairwise genetic distances between the different mtDNA clades within each species. However, this variance in gene divergence could also be due to stochasticity and different levels of ancestral polymorphism within each ancestral population [47,48]. The degree to which gene divergence predates population divergence can be substantial when the effective population size (*Ne*) of the common ancestor is large relative to the divergence time [47]. In fact, recent studies emphasize the need for model-based approaches to test the effects of shared historical factors in species/clades divergence [49,50]. Moreover, with just a single non-recombining locus, and especially if reciprocal monophyly has been achieved, it is not possible to differentiate between variance in sequence divergence due to divergence times and that resulting from coalescence. Given the different levels of intraspecific genetic divergence seen at the mtDNA, very different long-term population sizes would have to be assumed for each species to make them compatible with a synchronous north–south split. Multiple unlinked loci are extremely useful in addressing this question [47,51], as the expected variance in pairwise genetic distances from unlinked nuclear loci is directly proportional to the ancestral *Ne*, thereby providing information on coalescent processes in ancestral populations and reducing confidence intervals surrounding estimated divergence times eg. [49]. Accordingly, we estimated higher current and ancestral population sizes within *P. sundbergi* than within *P. astriata*. Interestingly, these estimates oppose current census sizes for both species, especially in the case of *P. sundbergi* populations of the northern islands [43,52], possibly indicating wide demographic changes across the evolutionary history of these species.

### Eco-morphological divergence

Assuming a simultaneous divergence between both the northern and southern clades in each species leads us to hypothesize that the different ecological/habitat conditions in the different island groups had a predominant role on the different degrees of body-size divergence within each island group. As shown with body size measurements from Gardner [32] and Radtkey [8], the mean difference in snout-vent length (SVL) between *P. sundbergi* and *P. astriata* within the northern group (Praslin, La Digue, and associated islands) is significantly higher than the corresponding difference in the southern group. Gardner [32] demonstrated the importance of body size in resource use with observations of the differential use of palm tree pollen and nectar. Palms from the genera *Lodoicea*, *Deckenia* and *Cocos* all produce male inflorescences that are actively defended for highly nutritious resources, and in all reported cases the largest individual displaced smaller individuals, regardless of the intruding species [32]. This author further suggested that the large body size of *P. sundbergi* in the northern group likely evolved through interference competition associated with the differential distribution of palm trees. More specifically, he argued that because *Lodoicea maldivica* (the larger palm species) occurs only in the northern islands of Praslin and Curieuse, the selection pressure necessary to drive the evolution of larger body size was absent in the southern group. Radtkey [8] discarded this hypothesis arguing that competition in *Cocos nucifera* plantations was equally intense, and that this palm, along with *Deckenia*, was distributed throughout all the Seychelles. Nevertheless, *L. maldivica* (native only to the northern islands of Praslin and Curieuse), was the dominant tree on these islands, occurring across a broad range of habitats from the coast to the uplands, prior to human colonization [11] and recent data highlights the clear association of *P. sundbergi* with adult *L. maldivica* males in Praslin [43]. Harmon et al. [44] investigating the competition and community structure within *Phelsuma* from the Mascarene islands also found that one of their unique characteristics compared to other diurnal arboreal lizards is the fact that they partition the habitat between palms and non-palm trees. They also reported that interspecific interactions exist and that species rapidly react to other species presence or removal shifting their habitat use. As argued by Gardner [32], it is therefore possible that in palm forests selection will favour adults with large body-size as they will be able to defend inflorescences successfully, ensuring a constant food supply. In fact, all large species of this genus in the Mascarenes are also associated with potentially defensible items, in which large size may be advantageous [32]. In other habitats small size is probably more effective.

All these aspects become relevant given our divergence time estimates and warrant further investigation on the ecological determinants of eco-morphological diversification within *Phelsuma* in the Seychelles. According to our results, the intermediate size of *P. sundbergi* in the southern group is not the result of its evolution as a solitary species to use a wider resource distribution; rather other variables should be considered. Species are likely partitioning their habitat differently within each island, which leads to different outcomes in terms of selection and body size evolution. The low overlap of structural niche categories shows for example that *P. astriata* from Praslin and Silhouette use different parts of the tree [32]. For example, on Silhouette *P. astriata* are more abundant than *P. sundbergi* in coconut plantations, where they use a similar microhabitat to *P. sundbergi* on Praslin. In Silhouette habitat segregation is considerable (with *P. astriata* reaching larger sizes than *P. sundbergi* and dominating nectiferous resources) while in Mahé species seem to co-exist without obvious resource partitioning, although some segregation is possible (*P. astriata* seems to be much less abundant at the medium perch height where *P. sundbergi* is most frequently observed; S. Rocha, pers. obs). Further investigation on habitat use and niche partitioning within both species across island groups will be determinant for the understanding of the role of the habitat and interspecific competition in the microevolutionary patterns within this group.

## Conclusions

Geographic patterns of genetic variation within *Phelsuma* species from the Seychelles support current taxonomy, with the taxonomic status of *P. astriata* population from Fregate remaining undetermined. The data are also congruent with the very recent origin of all outer islands populations. Despite extensive sympatric distributions, no gene flow seems to occur between the two species, whose divergence most probably pre-dates the Pleistocene. The genetic structure within each species is well correlated with geography, with the main mtDNA clades (subspecies) being distributed exclusively across northern and southern islands. Such differentiation is consistent with isolation caused by oscillating sea levels, which also seems to have allowed post-divergence gene flow within both species. The divergence between the main (northern and southern) clades within each species seems to have been approximately simultaneous, and highlights the importance of studying the potential role of habitat and ecological factors in microevolutionary patterns within this group.

## Methods

### Sampling and molecular data collection

Tissue samples (tail tips) of *Phelsuma sundbergi* and *P. astriata,* covering almost their entire distribution were collected and stored in 100% ethanol. Sampling spanned a 4-year period (2005–2008) with sampling locations not repeated, thus the likelihood of having sampled the same individual more than once is minimal. No vouchers were collected. DNA extraction followed standard salt or phenol-chloroform protocols [53,54]. Cytochrome-*b* (Cyt-*b*) was amplified in 126 *P. sundbergi* individuals and 73 *P. astriata* individuals, comprising all recognized morphological variation within each species (Figure 1). Between one and ten individuals per island per species were genotyped for four additional nuclear markers (see Additional file 1).

Primers for Cyt-*b* were CBL14753 [55] and CB3H [56] with amplification conditions as given by Rocha et al. [30]. Phosducin (*PDC*) amplification was made with primers and conditions from Gamble et al. [57], recombination-activating gene 2 (*Rag-2*) as in Rocha et al. [30], melanocortin-1 receptor (*MC1R*) and intron 61 of the reelin gene (*RELNint61*) using primers and conditions from Pinho et al. [58]. No evidences of multiple copies (size variation, modifications of reading frame) were found for any fragment. Sequences were determined by a commercial facility (Macrogen, Seoul, Korea). For most individuals we sequenced both strands of nuclear fragments to assure double peaks were real and not base calling errors.

Sequences were aligned manually using BioEdit [59]. For the nuclear genes, the haplotype phases were resolved using two complementary methods: first, for sequences that were heterozygous for insertions or deletions (some individuals for the *RELNint61* fragment), we used the method described by Flot et al. [60], after which we applied the Bayesian algorithm implemented in PHASE software [61] using the known phases of haplotypes determined by the previous method, when available. For the remaining genes PHASE was applied directly to the datasets. We considered all positions for which phases were resolved with a posterior probability higher than 0.8 (though the majority of them were actually resolved with 1 or > 0.9 probability). Remaining positions were coded as missing data (N) or with ambiguity codes for subsequent sequence analysis as appropriate, but complete individuals were never excluded. We performed a minimum of three runs of PHASE for each dataset and checked phase calls for consistency. Sequence accession numbers are KC347737-KC348370. All sequence files used in downstream analyses can also be downloaded from the DRYAD database (doi:http://10.5061/dryad.6q3t1).

### Genetic variability, neutrality tests and gene genealogies estimation

To assess how genetic variability was distributed across and within species we calculated a series of summary statistics for each marker and putative subspecies. As only three individuals of *P. astriata* from the Fregate population were collected (the “intermediate form” sensu [31]), summary statistics were not computed in this case.

For each locus, we calculated the number of haplotypes, the number of segregating sites, haplotype diversity, nucleotide diversity π, and population mutation parameter θ [62]. We tested for non-neutral evolution by computing Tajima’s D [63] and further tested each population for signals of demographic expansions by calculating Fu’s Fs [64] and R_2_[65]. We tested significance of the results of the three tests using 10,000 coalescent simulations. All these analyses were conducted in DnaSP v5 [66].

We used MrBayes v3.1.2 [67] to obtain an estimate of the *Cytb* phylogeny (BI). The dataset was reduced to single haplotypes using ALTER [68] and the best fitting model estimated using jModeltest [69] and PhyML [70], under the AICc criteria [71,72]. Two runs of 11 million generations with default heating parameters were performed. Convergence and congruence across runs were assessed using AWTY [73]. MEGA v4 [74] was used to estimate the mean distance between clades (Nei’s Da). Median-joining networks [75] with MP posterior optimization [76] were constructed using NETWORK v4.510 [77] and used to illustrate the relationships between nuclear and mitochondrial haplotypes (applied separately for each species in the latter).

### Testing the asynchronous colonization hypothesis: estimates of divergence times and gene flow under the isolation-with-migration model

Based on the results on population structure, we fitted a subset of each species data to a two-populations model (the northern and southern clades of each species), and used the isolation-with-migration (IM) model as implemented in the program IMa2 [78,79] to estimate the six parameters involved: ancestral and current population sizes (*θ*_*A*_, *θ*_*1*_, and *θ*_*2*_), migration in both directions (*m*_*1*_ and *m*_*2*_) and time since the population split (*T*). One individual of *P. sundbergi longinsulae* from Mahé island that carried a haplotype that clustered within *P. sundbergi sundbergi* haplotypes from Praslin was excluded from the analysis, as it could possibly reflect an anthropogenic introduction (see results and discussion). Within *P. astriata*, the Fregate population (N = 3) was also excluded due to its intermediate position relative to the other two groups of haplotypes.

The HKY [80] model was used for the mtDNA dataset and also for *Rag-2*, *RELNint61* and *MC1R* nuclear gene fragments, where homoplasic positions were detected, and the IS model [81] for *PDC*. Prior bounds were first selected based on the summary statistics and optimized and adjusted in a few preliminary runs, to ensure that prior distributions covered the whole range of possible values for that parameter. Multiple subsequent runs of each dataset were then performed and effective sample size of parameters (ESS) and trend plots examined to assure proper mixing and convergence. The significance of the estimated migration rates was measured by the LLR test of Nielsen and Wakeley [82]. A substitution rate of 1% per lineage per Myr (mtDNA, see below) and a generation time of one year, as approximately estimated by Gardner [32] for *P. astriata* (9 months to attain maturity and approximately 2.5 months egg incubation time) were used to convert parameter estimates into biologically meaningful quantities.

Additionally, to obtain an estimate of the divergence time between *P. sundbergi* and *P. astriata*, and to test for the existence of post-divergence gene flow across species, we also fitted the IM model to the whole dataset, treating each species as a separate population.

Because IMa2 assumes no intragenic recombination or selection we tested each locus for recombination using the pairwise homoplasy index [83] implemented in PhiPack [84], and for selection performing multiple loci HKA tests [85] using SITES and HKA [86].

### Species-tree inference

To obtain a multilocus perspective of the diversification within the whole clade, we employed the Bayesian MCMC method from Heled and Drummond [87], *BEAST, implemented in BEASTv1.6.1 [88]. This method makes use of the multispecies coalescent model to co-estimate the multiple gene trees and the “species-tree” they are embedded within, together with divergence times and effective population sizes of extant and ancestral species. Especially at this shallow level of divergence, it is likely that multiple processes such as demography and incomplete lineage sorting influence the gene genealogies and the implementation of a method that properly accommodates this variation, rather than a concatenation approach, becomes especially relevant [89,90] and references therein. A minimum of two individuals per “species” (the term species not necessarily referring to the taxonomic rank but to any diverging “population structure” – in our case the five subspecies) per loci is needed, while additional individuals increase accuracy [87]. We therefore constructed a dataset with a subsample of our individuals, using some of those that were genotyped for the five genes: 19 *P. sundbergi longinsulae*, 8 *P. sundbergi sundbergi*, 5 *P. sundbergi ladiguensis*, 20 *P. astriata astriata*, 22 *P. astriata semicarinata* and finally 2 individuals from *P. astriata* from Fregate (Additional file 1).

We estimated the appropriate model of evolution for each fragment as previously and implemented it choosing the most similar within the ones available in BEASTv1.6.1, with all related parameters (gamma; proportion of invariant sites, transition/transversion rates) being co-estimated along the runs. The multispecies coalescent allows for gene-specific mutation rates. Throughout, the substitution rate of the mitochondrial locus was set to a normal distribution prior of mean 0.01 (~1% per lineage per Myr) and standard deviation of 0.0027, as in [91]. Similar rates have been consistently recovered for mtDNA fragments of other reptile and amphibian groups at the intrageneric level, ranging from 0.99% in *Hydromantes*[92], to 1.35% in lacertids [93] and references therein], estimates available from calibrated trees for gekkonid falling within this range [94]; 1, 15% for *Hemidactylus*). Rate estimates around 1% per lineage per million year were also obtained by Chapple et al. [95] (mean 1.01%; David Chapple *pers. comm*) and Cox et al. [96] (S. Carranza *pers comm*) and the range 1.15-1.35% used also by Miralles and Carranza [97]. In the absence of any available external calibration, we believe that our rate distribution prior, which encompasses these values, is appropriate to obtain an approximate estimate of the time-scale of the diversification of this group (although see Discussion for the possibility of faster substitution rates). Nuclear rates were co-estimated along the run, relative to the mitochondrial one. An uncorrelated relaxed clock model was assumed for the *Cytb* dataset, whereas a strict clock was assumed for the nuclear gene fragments given their low variability (causing runs under relaxed clock models to fail to converge). We ran the dataset under both a Yule and a birth-death prior for the species-tree and a population size model of continuous growth and constant root (as recommended by the authors; J. Heled, *pers. comm*.). Default values were used for all other parameters. Multiple runs of 100–500 million generations were performed and sampled at appropriate intervals to obtain 10,000 final samples (trees). To test the influence of the priors on the posterior estimates, an additional run of identical length was made without data, sampling only from the prior. Tracer v1.5 was used to visualize the results of each run, to check the effective sample size of each parameter and chose appropriate burnin values [98]. After discarding the burnin samples, a consensus species tree (Maximum Clade Credibility tree), mean node heights and supports were obtained using TreeAnnotator v1.6.1 [88] and visualized using FigTree v1.3.1 [99].

## Competing interests

The authors declare that they have no competing interests.

## Authors’ contributions

SR and DJH collected the samples in the field. SR performed the laboratorial work, analysed the data and drafted the manuscript. SR, DJH and DP devised the analytical strategy. DJH and DP provided critical corrections and comments on the paper. All authors read and approved the final manuscript.

## Supplementary Material

Additional file 1 List of all analysed specimens with details on geographic origin and Genbank numbers. Click here for file

## References

[B1] Mittermeier RA Robles Gil P Hoffman J Pilgrim J Brooks T Mittermeier CG Lamoreaux J da Fonseca GAB Hotspots Revisited: Earth’s Biologically Richest and Most Endangered Terrestrial Ecoregions 2005 Chicago: University of Chicago Press

[B2] Cheke AS Hume JP Lost Land of the Dodo. An Ecological History of Mauritius, Réunion & Rodrigues 2008 London: Yale University Press

[B3] Gerlach J Terrestrial and freshwater vertebrates of the Seychelles islands 2007 Leiden: Backhuys Publishers

[B4] Hawlitschek O Brückmann B Berger J Green K Glaw F Integrating field surveys and remote sensing data to study distribution, habitat use and conservation status of the herpetofauna of the Comoro Islands Zookeys 2011 144 21 78 10.3897/zookeys.144.1648 22207785 PMC3233692

[B5] Yoder AD Nowak MD Has vicariance or dispersal been the predominant biogeographic force in madagascar? only time will tell Annu Rev Ecol Evol Syst 2006 37 405 431 10.1146/annurev.ecolsys.37.091305.110239

[B6] Warren BH Strasberg D Bruggemann JH Prys-jones RP Cladistics Why does the biota of the madagascar region have such a strong asiatic flavour? Cladistics 2010 26 526 538 10.1111/j.1096-0031.2009.00300.x 34875766

[B7] Agnarsson I Kuntner M Anamthawat-Jónsson K The generation of a biodiversity hotspot: biogeography and phylogeography of the western indian ocean islands Current topics in phylogenetics and phylogeography of terrestrial and aquatic systems 2012 Rijeka: In Tech Publishers 33 82 ISBN 978-953-51-0217-5

[B8] Radtkey RR Adaptive radiation of Day-geckos (Phelsuma) in the seychelles archipelago: a phylogenetic analysis Evolution 1996 50 604 623 10.2307/2410835 28568942

[B9] Gerlach J Snails of the genus Pachnodus (Mollusca; Gastropoda; Enidae): their origins and evolution J Biogeogr 1999 26 251 255 10.1046/j.1365-2699.1999.00259.x

[B10] Silva A Harris DJ Rocamora G Dufrenne A Gerlach J Rocha S Assessment of mtDNA genetic diversity within the terrapins *Pelusios subniger* and *Pelusios castanoides* across the Seychelles islands Amphibia-Reptilia 2010 31 583 588 10.1163/017353710X524723

[B11] Fleischer-Dogley F Kettle CJ Edwards PJ Ghazoul J Määttänen K Kaiser-Bunbury CN Morphological and genetic differentiation in populations of the dispersal-limited coco de mer (*Lodoicea maldivica*): implications for management and conservation Divers Distrib 2011 17 235 243 10.1111/j.1472-4642.2010.00732.x

[B12] Daniels SR Reconstructing the colonisation and diversification history of the endemic freshwater crab (*Seychellum alluaudi*) in the granitic and volcanic Seychelles Archipelago Mol Phylogenet Evol 2011 61 534 542 10.1016/j.ympev.2011.07.015 21824522

[B13] Rocha S Harris DJ Posada D Cryptic diversity within the endemic prehensile-tailed gecko Urocotyledon inexpectata across the Seychelles Islands: patterns of phylogeographical structure and isolation at the multilocus level Biol J Linn Soc 2011 104 177 191 10.1111/j.1095-8312.2011.01710.x

[B14] Braithwaite CJR Stoddart DR Scientific studies in the Seychelles Biogeography of the Seychelles Islands 1984 The Hague: Dr W Junk 1 16

[B15] Plummer PS Amin M, Willetts D, Skerett A Planet Aldabra Aldabra world heritage site 1995 Nairobi: Camerapix Publishers International 49 70

[B16] Colonna MJ Casanova J Dullo WC Camoin G Sea level changes and ∂18O record for the past 34,000 yr from Mayotte reef, Indian Ocean Quaternary Res 1996 46 335 339 10.1006/qres.1996.0071

[B17] Siddall M Rohling EJ Almogi-Labin A Hemleben C Meischner D Schmelzer I Smeed DA Sea-level fluctuations during the last glacial cycle Nature 2003 423 19 24 10.1038/nature01690 12815427

[B18] Miller KG Kominz MA Browning JV Wright JD Mountain GS Katz ME Sugarman PJ Cramer BS Christie-blick N Pekar SF The phanerozoic record of global Sea-level change Science 2005 310 1293 1298 10.1126/science.1116412 16311326

[B19] Inger RF Stuart BL Iskandar DT Systematics of a widespread Southeast Asian frog, Rana chalconota (Amphibia: Anura: Ranidae) Zool J Linn Soc-London 2009 155 123 147 10.1111/j.1096-3642.2008.00440.x

[B20] Siler CD Oaks JR Esselstyn JA Diesmos AC Brown RM Phylogeny and biogeography of Philippine bent-toed geckos (Gekkonidae: *Cyrtodactylus*) contradict a prevailing model of Pleistocene diversification Mol Phylogenet Evol 2010 55 699 710 10.1016/j.ympev.2010.01.027 20132898

[B21] Scott H General conclusions regarding the insect fauna of the Seychelles and adjacent islands Trans Linn Soc Lond 2nd series (Zoology) 1933 19 307 391 10.1111/j.1096-3642.1933.tb00131.x

[B22] Cheke AS Stoddart DR Lizards of the Seychelles Biogeography and ecology of the Seychelles Islands 1984 The Hague: Dr. W. Junk 245 258

[B23] Gerlach J van Bruggen AC Streptaxidae (Mollusca: Gastropoda: Pulmonata) of the Seychelles Islands, Western Indian Ocean Zool Verh Leiden 1999 328 1 60

[B24] Nussbaum RA Wu SH Distribution, variation, and systematics of the Seychelles treefrog, *Tachycnemis seychellensis* (Amphibia: Anura: Hyperoliidae) J Zool 1995 236 383 406 10.1111/j.1469-7998.1995.tb02720.x

[B25] Gardner AS The systematics of the *Phelsuma madagascariensis* species group of day geckos (Reptilia: Gekkonidae) in the Seychelles Zool J Linn Soc-London 1987 91 93 105 10.1111/j.1096-3642.1987.tb01724.x

[B26] Nussbaum RA Stoddart DR The amphibians of the Seychelles Biogeography and ecology of the Seychelles Islands 1984 The Hague: Dr. W. Junk 378 415

[B27] Van Der Meijden A Boistel R Gerlach J Ohler A Vences M Meyer A Molecular phylogenetic evidence for paraphyly of the genus Sooglossus, with the description of a new genus of Seychellean frogs Biol J Linn Soc 2007 91 347 359 10.1111/j.1095-8312.2007.00800.x

[B28] Rocha S Rösler H Gehring P Glaw F Posada D Harris DJ Vences M Phylogenetic systematics of day geckos, genus *Phelsuma*, based on molecular and morphological data (Squamata: Gekkonidae) Zootaxa 2010 28 1 28

[B29] Harmon LJ Melville J Larson A Losos JB The role of geography and ecological opportunity in the diversification of day geckos (*Phelsuma*) Syst Biol 2008 57 562 573 10.1080/10635150802304779 18686194

[B30] Rocha S Vences M Glaw F Posada D Harris DJ Multigene phylogeny of Malagasy day geckos of the genus *Phelsuma* Mol Phylogenet Evol 2009 52 530 537 10.1016/j.ympev.2009.03.032 19362158

[B31] Cheke AS *Phelsuma* GRAY 1825 in the Seychelles and neighbouring islands: a re-appraisal of their taxonomy and description of two new forms (Reptilia: Sauria: Gekkonidae) Senckenberg biol 1982 62 181 198

[B32] Gardner AS The evolutionary ecology and population systematics of day-geckos genus Phelsuma in the Seychelles PhD thesis 1984 Aberdeen: University of Aberdeen

[B33] Gardner AS Morphological evolution in the day gecko *Phelsuma sundbergi* in the Seychelles: a multivariate study Biol J Linn Soc 1986 29 223 244 10.1111/j.1095-8312.1986.tb01774.x

[B34] Borner AR Minuth W On the taxonomy of the Indian Ocean lizards of the Phelsuma madagascariensis species group (Reptilia: Geckonidae) J Bomb Nat Hist Soc 1985 81 243 281

[B35] Mertens R Die nichmadagassischen arten and unterarten der geckonengattung *Phelsuma* Senckenberg biol 1966 47 85 110

[B36] Meiri S Size evolution in island lizards Global Ecol Biogeogr 2007 16 702 708 10.1111/j.1466-8238.2007.00327.x

[B37] Dayan T Simberloff D Ecological and community-wide character displacement: the next generation Ecol Lett 2005 8 875 894 10.1111/j.1461-0248.2005.00791.x

[B38] Martin PR Montgomerie R Lougheed SC Rapid sympatry explains greater color pattern divergence in high latitude birds Evolution 2010 64 336 347 10.1111/j.1558-5646.2009.00831.x 19744123

[B39] Melo M Warren BH Jones PJ Rapid parallel evolution of aberrant traits in the diversification of the Gulf of Guinea white-eyes (Aves, Zosteropidae) Mol Ecol 2011 20 4953 4967 10.1111/j.1365-294X.2011.05099.x 21599770

[B40] Schluter D The ecology of adaptive radiation 2000 New York: Oxford University Press

[B41] Ackerly DD Schwilk DW Webb CO Niche evolution and adaptive radiation: testing the order of trait divergence Ecology 2006 87 S50 S61 10.1890/0012-9658(2006)87[50:NEAART]2.0.CO;2 16922302

[B42] Losos JB Adaptive radiation, ecological opportunity, and evolutionary determinism Am Nat 2010 175 623 639 10.1086/652433 20412015

[B43] Noble T Bunbury N Kaiser-Bunbury CN Bell DJ Ecology and co-existence of two endemic day gecko (*Phelsuma*) species in Seychelles native palm forest J Zool 2011 283 73 80 10.1111/j.1469-7998.2010.00751.x

[B44] Harmon JL Harmon LL Jones CG Competition and community structure in diurnal arboreal geckos (genus *Phelsuma*) in the Indian Ocean Oikos 2007 116 1863 1878

[B45] Carranza S Arnold EN A review of the geckos of the genus *Hemidactylus* (Squamata: Gekkonidae) from Oman based on morphology, mitochondrial and nuclear data, with descriptions of eight new species Zootaxa 2012 3378 1 95

[B46] Strasburg JL Rieseberg LH How robust are “isolation with migration” analyses to violations of the im model? A simulation study Mol Biol Evol 2010 27 297 310 10.1093/molbev/msp233 19793831 PMC2877552

[B47] Edwards SV Beerli P Perspective: gene divergence, population divergence, and the variance in coalescence time in phylogeographic studies Gene 2000 54 1839 1854 10.1111/j.0014-3820.2000.tb01231.x 11209764

[B48] Hickerson MJ Dolman G Moritz C Comparative phylogeographic summary statistics for testing simultaneous vicariance Mol Ecol 2006 15 209 223 16367841 10.1111/j.1365-294X.2005.02718.x

[B49] Hurt C Anker A Knowlton N A multilocus test of simoultaneous divergence across the isthmus of Panama using snapping shrimp in the genus *Alpheus* Evolution 2009 63 514 530 10.1111/j.1558-5646.2008.00566.x 19154357

[B50] Knowles LL Statistical phylogeography Annu Rev Ecol Evol Syst 2009 40 593 612 10.1146/annurev.ecolsys.38.091206.095702

[B51] Wakeley J Hey J Estimating ancestral population parameters Genetics 1997 145 847 855 9055093 10.1093/genetics/145.3.847 PMC1207868

[B52] Gerlach J Population and conservation status of the reptiles of the Seychelles islands Phelsuma 2008 16 31 48

[B53] Kocher TD Thomas WK Meyer A Edwards SV Paäbo S Villablanca FX Wilson AC Dynamics of mitochondrial DNA evolution in animals- amplification and sequencing with conserved primers P Natl Acad Sci USA 1989 86 6196 6200 10.1073/pnas.86.16.6196 PMC297804 2762322

[B54] Sambrook JE Fritsh EF Maniatis T Molecular cloning: a laboratory manual 1989 2 New York: Cold Spring Harbour Press

[B55] Austin JJ Arnold EN Jones CG Reconstructing an island radiation using ancient and recent DNA: the extinct and living day geckos (*Phelsuma*) of the Mascarene islands Mol Phylogenet Evol 2004 31 109 122 10.1016/j.ympev.2003.07.011 15019612

[B56] Palumbi SR Martin A Romano S McMillan WO Stice L Grabowski G The simple Fool’s guide to PCR, version 2.0 1991 Hawaii: Privately published

[B57] Gamble T Bauer AM Greenbaum E Jackman TR Evidence for Gondwanan vicariance in an ancient clade of gecko lizards J Biogeogr 2008 35 88 104

[B58] Pinho C Rocha S Carvalho BM Lopes S Mourão S Vallinoto M Brunes TO Haddad CFB Gonçalves H Sequeira F Ferrand N New primers for the amplification and sequencing of nuclear loci in a taxonomically wide set of reptiles and amphibians Conserv Genet Resour 2010 2 181 185

[B59] Hall TA BioEdit: a user friendly biological sequence alignment editor and analysis program for Windows 95/98/NT Nucleic Acids Symp 1999 41 95 98

[B60] Flot J-F Tillier J Samadi S Tillier S Phase determination from direct sequencing of length-variable DNA regions Mol Ecol Notes 2006 6 627 630 10.1111/j.1471-8286.2006.01355.x

[B61] Stephens M Smith NJ Donnelly P A new statistical method for haplotype reconstruction from population data Am J Hum Genet 2001 68 978 989 10.1086/319501 11254454 PMC1275651

[B62] Watterson GA On the number of segregating sites in genetical models without recombination Theor Popul Biol 1975 7 256 276 10.1016/0040-5809(75)90020-9 1145509

[B63] Tajima F Statistical method for testing the neutral mutation hypothesis by DNA polymorphism Genetics 1989 123 585 595 2513255 10.1093/genetics/123.3.585 PMC1203831

[B64] Fu YX Statistical tests of neutrality of mutations against population growth, hitchhiking and background selection Genetics 1997 147 915 925 9335623 10.1093/genetics/147.2.915 PMC1208208

[B65] Ramos-Onsins SE Rozas J Statistical properties of new neutrality tests against population growth Mol Biol Evol 2002 19 2092 2100 10.1093/oxfordjournals.molbev.a004034 12446801

[B66] Librado P Rozas J DnaSP v5: a software for comprehensive analysis of DNA polymorphism data Bioinformatics 2009 25 1451 1452 10.1093/bioinformatics/btp187 19346325

[B67] Ronquist F Huelsenbeck JP MrBayes 3: Bayesian phylogenetic inference under mixed models Bioinformatics 2003 19 1572 1574 10.1093/bioinformatics/btg180 12912839

[B68] Glez-Peña D Gómez-Blanco D Reboiro-Jato M Fdez-Riverola F Posada D ALTER: program-oriented conversion of DNA and protein alignments Nucleic Acids Res 2010 38 W14 W18 10.1093/nar/gkq321 20439312 PMC2896128

[B69] Posada D jModelTest: phylogenetic model averaging Mol Biol Evol 2008 25 1253 1256 10.1093/molbev/msn083 18397919

[B70] Guindon S Gascuel O A simple, fast, and accurate algorithm to estimate large phylogenies by maximum likelihood Syst Biol 2003 52 696 704 10.1080/10635150390235520 14530136

[B71] Sugiura N Further analysis of the data by akaike’ s information criterion and the finite corrections Commun Stat A - Theor 1978 7 13 26 10.1080/03610927808827599

[B72] Posada D Buckley TR Model selection and model averaging in phylogenetics: advantages of akaike information criterion and bayesian approaches over likelihood ratio tests Syst Biol 2004 53 793 808 10.1080/10635150490522304 15545256

[B73] Nylander JA Wilgenbusch JC Warren DL Swofford DL AWTY (are we there yet?): a system for graphical exploration of MCMC convergence in Bayesian phylogenetics Bioinformatics 2008 24 581 583 10.1093/bioinformatics/btm388 17766271

[B74] Kumar S Nei M Dudley J Tamura K MEGA: a biologist-centric software for evolutionary analysis of DNA and protein sequences Brief Bioinform 2008 9 299 306 10.1093/bib/bbn017 18417537 PMC2562624

[B75] Bandelt HJ Forster P Röhl A Median-joining networks for inferring intraspecific phylogenies Mol Biol Evol 1999 16 37 48 10.1093/oxfordjournals.molbev.a026036 10331250

[B76] Polzin T Daneshmand SV On steiner trees and minimum spanning trees in hypergraphs Oper Res Lett 2003 31 12 20 10.1016/S0167-6377(02)00185-2

[B77] NETWORK v4.510 Available from http://fluxus-engineering.com

[B78] Hey J Nielsen R Integration within the Felsenstein equation for improved Markov chain Monte Carlo methods in population genetics P Natl Acad Sci USA 2007 104 2785 2790 10.1073/pnas.0611164104 PMC1815259 17301231

[B79] Hey J Isolation with migration models for more than two populations Mol Biol Evol 2010 27 905 920 10.1093/molbev/msp296 19955477 PMC2877539

[B80] Hasegawa M Kishino H Yano T Dating of the human-ape splitting by a molecular clock of mitochondrial DNA J Mol Evol 1985 22 160 174 10.1007/BF02101694 3934395

[B81] Kimura M The number of heterozygous nucleotide sites maintained in a finite population due to steady flux of mutations Genetics 1969 61 893 903 5364968 10.1093/genetics/61.4.893 PMC1212250

[B82] Nielsen R Wakeley J Distinguishing migration from isolation: a Markov chain Monte Carlo approach Genetics 2001 158 885 896 11404349 10.1093/genetics/158.2.885 PMC1461674

[B83] Bruen TC Philippe H Bryant D A simple and robust statistical test for detecting the presence of recombination Genetics 2006 172 2665 2681 16489234 10.1534/genetics.105.048975 PMC1456386

[B84] Bruen T Bruen T PhiPack: PHI test and other tests of recombinationavailable at http://www.maths.otago.ac.nz/~dbryant/software.html

[B85] Hudson RR Kreitman M Aguadé M A test of neutral molecular evolution based on nucleotide data Genetics 1987 116 153 159 3110004 10.1093/genetics/116.1.153 PMC1203113

[B86] Hey J SITES and HKAavailable at http://genfaculty.rutgers.edu/hey/software

[B87] Heled J Drummond AJ Bayesian inference of species trees from multilocus data Mol Biol Evol 2010 27 570 580 10.1093/molbev/msp274 19906793 PMC2822290

[B88] Drummond AJ Rambaut A BEAST: Bayesian evolutionary analysis by sampling trees BMC Evol Biol 2007 7 214 10.1186/1471-2148-7-214 17996036 PMC2247476

[B89] Edwards SV Is a new and general theory of molecular systematics emerging? Evolution 2009 63 1 19 10.1111/j.1558-5646.2008.00549.x 19146594

[B90] Leaché AD Species trees for spiny lizards (genus Sceloporus): identifying points of concordance and conflict between nuclear and mitochondrial data Mol Phylogenet Evol 2010 54 162 171 10.1016/j.ympev.2009.09.006 19751840

[B91] Paulo OS Pinheiro J Miraldo A Bruford MW Jordan WC Nichols RA The role of vicariance vs. dispersal in shaping genetic patterns in ocellated lizard species in the western Mediterranean Mol Ecol 2008 17 1535 1551 10.1111/j.1365-294X.2008.03706.x 21928468

[B92] Carranza S Romano A Arnold EN Sotgiu G Biogeography and evolution of European cave salamanders, *Hydromantes* (Urodela: Plethodontidae), inferred from mtDNA sequences J Biogeogr 2008 35 724 738 10.1111/j.1365-2699.2007.01817.x

[B93] Carranza S Arnold EN Geniez P Roca J Mateo JA Radiation, multiple dispersal and parallelism in the skinks, *Chalcides* and *Sphenops* (Squamata: Scincidae), with comments on Scincus and Scincopus and the age of the Sahara Desert Mol Phylogenet Evol 2008 46 1071 1094 10.1016/j.ympev.2007.11.018 18276164

[B94] Arnold EN Vasconcelos R Harris DJ Mateo JA Carranza S Systematics, biogeography and evolution of the endemic *Hemidactylus* geckos (Reptilia, Squamata, Gekkonidae) of the Cape Verde Islands: based on morphology and mitochondrial and nuclear DNA sequences Zool Scr 2008 37 619 636 10.1111/j.1463-6409.2008.00351.x

[B95] Chapple DG Ritchie PA Daugherty CH Origin, diversification, and systematics of the New Zealand skink fauna (Reptilia: Scincidae) Mol Phylogenet Evol 2009 52 470 487 10.1016/j.ympev.2009.03.021 19345273

[B96] Cox SC Carranza S Brown RP Divergence times and colonization of the Canary Islands by *Gallotia* lizards Mol Phylogenet Evol 2010 56 747 757 10.1016/j.ympev.2010.03.020 20307675

[B97] Miralles A Carranza S Systematics and biogeography of the Neotropical genus Mabuya, with special emphasis on the Amazonian skink *Mabuya nigropunctata* (Reptilia, Scincidae) Mol Phylogenet Evol 2010 54 857 869 10.1016/j.ympev.2009.10.016 19874906

[B98] Rambaut A Drummond AJ Tracerv1.5 2007 Available from http://beast.bio.ed.ac.uk/Trace

[B99] Rambaut A FigTreev1.3.1 2009 Available from http://tree.bio.ed.ac.uk/software/figtree/

