# Supplementary material for: Phylogeography and diversification history of the day-gecko genus Phelsuma in the Seychelles islands
Source: BMC Evol Biol. 2013 Jan 5;13:3. doi: 10.1186/1471-2148-13-3 (PMC3598968; doi:10.1186/1471-2148-13-3)
Supplement: Additional file 1 — List of all analysed specimens with details on geographic origin and Genbank numbers. [file 1471-2148-13-3-S1.doc]

Additional file 1 - Locality information for the studied specimens and Genbank accession numbers. For detailed geographical coordinates refer to Location ID in Rocha et al. (2009). Individuals used for species-tree reconstruction are marked with an *

|  |  |  | **Accession Numbers** | | | | |
| --- | --- | --- | --- | --- | --- | --- | --- |
| **Species** | **Individual** | **Locality** | *Cytb* | *Rag-2* | *Relnint61* | *MC1R* | *PDC* |
| *P. sundbergi longinsulae* | MA1 | 68 Airport, Mahé | KC347737 | FJ830318 |  |  |  |
| *P. sundbergi longinsulae* | MA7 * | 68 Airport, Mahé | KC347738 | KC348115 | GU180996 | GU180950 | KC348023 |
| *P. sundbergi longinsulae* | MA8 | 68 Airport, Mahé | KC347739 | KC348115 | KC348280 | KC347936 |  |
| *P. sundbergi longinsulae* | MA9 | 25 Beau Vallon (beach), Mahé | KC347740 |  |  |  |  |
| *P. sundbergi longinsulae* | MA10 * | 25 Beau Vallon (beach), Mahé | KC347741 | KC348115 | KC348281/2^c^ | KC347937 | KC348022 |
| *P. sundbergi longinsulae* | MA20 | 68B Airport, Mahé | KC347742 |  |  |  |  |
| *P. sundbergi longinsulae* | 2MA3 | 22 Glacis, Mahé | KC347743 | KC348115 |  |  |  |
| *P. sundbergi longinsulae* | 2MA4 | 22 Glacis, Mahé | KC347744 |  |  |  |  |
| *P. sundbergi longinsulae* | 2MA13 | 20 North2, Mahé | KC347745 | KC348115 |  |  |  |
| *P. sundbergi longinsulae* | 2MA14a | 20 North2, Mahé | KC347746 |  |  |  |  |
| *P. sundbergi longinsulae* | 2MA14 | 20 North2, Mahé | KC347747 |  |  |  |  |
| *P. sundbergi longinsulae* | 2MA15 | 20 North2, Mahé | KC347748 |  |  |  |  |
| *P. sundbergi longinsulae* | 2MA20 | 18 NorthEast Point, Mahé | KC347749 | KC348120 |  |  |  |
| *P. sundbergi longinsulae* | 2MA26 | 40 Morne Blanc, Mahé | KC347750 | KC348121 |  |  | KC348024 |
| *P. sundbergi longinsulae* | 2MA39 | 29 Mare aux Cochons 2, Mahé | KC347751 | KC348122 |  |  | KC348025 |
| *P. sundbergi longinsulae* | 2MA40 | 29 Mare aux Cochons 2, Mahé | KC347752 | KC348123 |  |  |  |
| *P. sundbergi longinsulae* | 2MA42 | 29 Mare aux Cochons 2, Mahé | KC347753 |  |  |  |  |
| *P. sundbergi longinsulae* | 2MA45 | 26 Beau Vallon (cross), Mahé | KC347754 | KC348124 |  |  |  |
| *P. sundbergi longinsulae* | 2MA46 * | 65 La Reserve 1, Mahé | KC347755 | KC348125 | KC348286 |  | KC348026 |
| *P. sundbergi longinsulae* | 2MA47 | 65 La Reserve 1, Mahé | KC347756 |  |  |  |  |
| *P. sundbergi longinsulae* | 2MA54 | 66 La Reserve 2, Mahé | KC347757 | KC348126 |  |  |  |
| *P. sundbergi longinsulae* | 2MA64 | 55 Anse aux Forbans, Mahé | KC347758 | KC348127 |  |  |  |
| *P. sundbergi longinsulae* | 2MA65 | 55 Anse aux Forbans, Mahé | KC347759 |  |  |  |  |
| *P. sundbergi longinsulae* | 2MA66 | 55 Anse aux Forbans, Mahé | KC347760 |  |  |  |  |
| *P. sundbergi longinsulae* | 2MA67 | 55 Anse aux Forbans, Mahé | KC347761 |  |  |  |  |
| *P. sundbergi longinsulae* | 2MA77 | 67 Grand Anse, Mahé | KC347762 | KC348128 | KC348283 | KC347938 |  |
| *P. sundbergi longinsulae* | 2MA81 * | 35 Port Glaud, Mahé | KC347763 | KC348129 | KC348284 | KC347939 | KC348027 |
| *P. sundbergi longisulae* | 2MA25 | 40 Morne Blanc, Mahé | KC347764 | KC348130 |  |  |  |
| *P. sundbergi longinsulae* | 38721 | 16 Mont Fleuri, Victoria, Mahé | KC347765 | KC348131 |  |  |  |
| *P. sundbergi longinsulae* | 3MA17 | 62 Anse a la Mouche, Mahé | KC347766 | KC348132 |  | KC347940 | KC348028 |
| *P. sundbergi longinsulae* | 3MA18 | 62 Anse a la Mouche, Mahé | KC347767 |  |  |  |  |
| *P. sundbergi longinsulae* | 3MA21 | 62 Anse a la Mouche, Mahé | KC347768 |  |  |  |  |
| *P. sundbergi longinsulae* | 3MA22 | 62 Anse a la Mouche, Mahé | KC347769 |  |  |  |  |
| *P. sundbergi longinsulae* | 3MA23 | 62 Anse a la Mouche, Mahé | KC347770 |  |  |  |  |
| *P. sundbergi longinsulae* | 3MA24 | 62 Anse a la Mouche, Mahé | KC347771 | KC348133 |  | KC347941 | KC348029 |
| *P. sundbergi longinsulae* | 3MA33 | 66 La Reserve 2, Mahé | KC347772 | KC348134 |  |  |  |
| *P. sundbergi longinsulae* | 3MA42 * | 59 Baie Lazare, Mahé | KC347773 | KC348135 | KC348285 | KC347942 | KC348030 |
| *P. sundbergi longinsulae* | 3MA46 | 59 Baie Lazare, Mahé | KC347774 |  |  |  |  |
| *P. sundbergi longinsulae* | 3MA48 | 59 Baie Lazare, Mahé | KC347775 |  |  |  |  |
| *P. sundbergi longinsulae* | 3MA49 | 59 Baie Lazare, Mahé | KC347776 |  |  |  |  |
| *P. sundbergi longinsulae* | 3MA50 | 59 Baie Lazare, Mahé | KC347777 | KC348136 |  |  |  |
| *P. sundbergi longinsulae* | 3MA78 * | 17 Port, Victoria, Mahé | KC347778 | KC348137 | KC348287 | KC347943 | KC348031 |
| *P. sundbergi longinsulae* | 3MA86 | 47 Trois Freres 2, Mahé | KC347779 | KC348138 |  |  |  |
| *P. sundbergi longinsulae* | 3MA94 | 25B Beau Vallon (beach), Mahé | KC347780 | KC348139 | KC348288 |  |  |
| *P. sundbergi longinsulae* | 3MA99 | 25B Beau Vallon (beach), Mahé | KC347781 |  |  |  |  |
| *P. sundbergi longinsulae* | 3MA101 | 24 Mare Anglaise, Mahé | KC347782 | KC348140 |  |  |  |
| *P. sundbergi longinsulae* | 3MA102 | 24 Mare Anglaise, Mahé | KC347783 |  |  |  |  |
| *P. sundbergi longinsulae* | 3MA108 * | 18 NorthEast Point, Mahé | KC347784 | KC348141 | KC348289 | KC347944 | KC348032 |
| *P. sundbergi longinsulae* | 3MA109 | 18 NorthEast Point, Mahé | KC347785 |  |  |  |  |
| *P. sundbergi longinsulae* | 3MA110 | 18 NorthEast Point, Mahé | KC347786 |  |  |  |  |
| *P. sundbergi longinsulae* | 3MA111 | 18 NorthEast Point, Mahé | KC347787 |  |  |  |  |
| *P. sundbergi longinsulae* | 3MA123 | 48 Brilliant, Mahé | KC347788 | KC348142 |  |  |  |
| *P. sundbergi longinsulae* | 3MA128 | 49 Cascade, Mahé | KC347789 |  |  |  |  |
| *P. sundbergi longinsulae* | 3MA129 | 49 Cascade, Mahé | KC347790 |  |  |  |  |
| *P. sundbergi longinsulae* | 3MA130 | 50 Anse aux Pins 1, Mahé | KC347791 | KC348143 |  |  |  |
| *P. sundbergi longinsulae* | 3MA135 | 34 Cap Ternay, Mahé | KC347792 | KC348144 |  |  |  |
| *P. sundbergi longinsulae* | 3MA137 | 16 Mont Fleuri, Victoria, Mahé | KC347793 | KC348145 |  |  |  |
| *P. sundbergi longinsulae* | 3MA145 | 44 Fairview, Mahé | KC347794 | KC348146 |  |  |  |
| *P. sundbergi longinsulae* | 3MA146 | 44 Fairview, Mahé | KC347795 |  |  |  |  |
| *P. sundbergi longinsulae* | 3MA147 | 17 Port, Victoria, Mahé | KC347796 | KC348147 |  |  |  |
| *P. sundbergi longinsulae* | 3MA151 | 31 Danzil, Mahé | KC347797 | KC348148 |  |  |  |
| *P. sundbergi longinsulae* | 3MA160 | 39 Tea Plantation, Mahé | KC347798 | KC348149 |  |  |  |
| *P. sundbergi longinsulae* | 3MA171 | 45 Copolia, Mahé | KC347799 | KC348150 |  |  |  |
| *P. sundbergi longinsulae* | 3MA178 | 36 Port Glaud 2, Mahé | KC347800 | KC348151 |  |  |  |
| *P. sundbergi longinsulae* | 3MA185 | 35 Port Glaud, Mahé | KC347801 | KC348152 |  |  |  |
| *P. sundbergi longinsulae* | 3MA196 | 64 Anse Boileau, Mahé |  | KC348153 |  |  |  |
| *P. sundbergi longinsulae* | 3MA197 | 64 Anse Boileau, Mahé | KC347802 |  |  |  |  |
| *P. sundbergi longinsulae* | 3MA202 | 67 Grand Anse, Mahé | KC347803 | KC348154 |  |  |  |
| *P. sundbergi longinsulae* | 3MA203 | 67 Grand Anse, Mahé | KC347804 | KC348155 |  |  |  |
| *P. sundbergi longinsulae* | 3MA204 | 51 Anse aux Pins 2, Mahé | KC347805 | KC348156 |  |  |  |
| *P. sundbergi longinsulae* | 3MA205 | 51 Anse aux Pins 2, Mahé | KC347806 |  |  |  |  |
| *P. sundbergi ssp.* | 3MA206^a^ | 52 Anse Royalle, Mahé | KC347807 | KC348157 | KC348290 | KC347945 |  |
| *P. sundbergi longinsulae* | CF1 * | 69 Cerf Island 1 | KC347808 | KC348158 | KC348291 | KC347946 | KC348033 |
| *P. sundbergi longinsulae* | CF9 * | 70 Cerf Island 2 | KC347809 | KC348159 | KC348292/3^c^ | KC347947 | KC348034 |
| *P. sundbergi longinsulae* | CF10 * | 70 Cerf Island 2 | KC347810 | KC348160 | KC348294 | KC347948 | KC348035 |
| *P. sundbergi longinsulae* | 7FG * | 114 Fregate | KC347849 | KC348174 |  | GU180952 | KC348044 |
| *P. sundbergi longinsulae* | 9FG * | 114 Fregate | KC347850 | KC348175 | KC348304/5^c^ |  | KC348045 |
| *P. sundbergi longinsulae* | 17FG | 114 Fregate |  | KC348176 | KC348306/7^c^ | KC347958 | KC348046 |
| *P. sundbergi longinsulae* | 18FG | 114 Fregate | KC347851 | KC348177 | GU181000 |  |  |
| *P. sundbergi longinsulae* | 22FG | 114 Fregate | KC347852 | KC348178 |  | KC347959 |  |
| *P. sundbergi longinsulae* | 34FG * | 114 Fregate | KC347853 | KC348179 | KC348303 | KC347960 | KC348048 |
| *P. sundbergi longinsulae* | 47FG | 114 Fregate | KC347854 | KC348180 |  | KC347961 | KC348049 |
| *P. sundbergi longinsulae* | 46FG | 114 Fregate | KC347855 | KC348181 | KC348308 |  |  |
| *P. sundbergi longinsulae* | 24FG | 114 Fregate | KC347856 | KC348182 |  |  | KC348047 |
| *P. sundbergi longinsulae* | PhSNorth | 115 North Island |  | KC348161 |  |  |  |
| *P. sundbergi longinsulae* | 7NORTH | 115 North Island | KC347811 | KC348162 | GU180997 | GU180951 |  |
| *P. sundbergi longinsulae* | 8NORTH * | 115 North Island | KC347812 | KC348163 | KC348295 | KC347949 | KC348036 |
| *P. sundbergi longinsulae* | 10NORTH | 115 North Island | KC347813 | KC348164 |  | KC347950 | KC348037 |
| *P. sundbergi longinsulae* | 11NORTH | 115 North Island | KC347814 | KC348165 | KC348296 | KC347951 | KC348038 |
| *P. sundbergi longinsulae* | 1SILH | 71 La Passe 1, Silhouette | KC347815 | KC348166 | KC348297/8^c^ | KC347952 |  |
| *P. sundbergi longinsulae* | 2SILH * | 71 La Passe 1, Silhouette | KC347816 | KC348167 | KC348299 | KC347953 | KC348039 |
| *P. sundbergi longinsulae* | 3SILH * | 71 La Passe 1, Silhouette | KC347817 | KC348168 | KC348300 | KC347954 | KC348040 |
| *P. sundbergi longinsulae* | 13SILH | 75 Anse Lascars, Silhouette | KC347818 | KC348169 |  | KC347955 |  |
| *P. sundbergi longinsulae* | 16SILH | 75 Anse Lascars, Silhouette | KC347819 | KC348170 |  |  |  |
| *P. sundbergi longinsulae* | 17SILH * | 75 Anse Lascars, Silhouette | KC347820 | KC348171 | KC348301 | KC347956 | KC348041 |
| *P. sundbergi longinsulae* | 26SILH * | 76 around GB rock, Silhouette | KC347821 | KC348172 | KC348302 | KC347957 | KC348042 |
| *P. sundbergi longinsulae* | 33SILH | 76 around GB rock, Silhouette | KC347822 | KC348173 |  |  | KC348043 |
|  |  |  |  |  |  |  |  |
| *P. sundbergi sundbergi* | PL5 * | 99 Anse Volbert, Praslin | KC347823 | KC348184 | GU180998 | KC347962 | KC348050 |
| *P. sundbergi sundbergi* | PL7 | 99 Anse Volbert, Praslin | KC347824 | KC348185 |  |  |  |
| *P. sundbergi sundbergi* | PL8 | 99 Anse Volbert, Praslin | KC347825 | KC348186 |  |  |  |
| *P. sundbergi sundbergi* | PL31 * | 110 Fonde de L'Anse, Praslin | KC347826 | KC348187 | KC348309 | KC347963 | KC348051 |
| *P. sundbergi sundbergi* | 38727 | 109 Grand Anse, Praslin | KC347827 | KC348188 | KC348310 | KC347964 |  |
| *P. sundbergi sundbergi* | 5PL * | 99 Anse Volbert, Praslin | KC347828 | KC348189 | KC348311 | GU180953 | KC348052 |
| *P. sundbergi sundbergi* | 6PL | 99 Anse Volbert, Praslin | KC347829 | KC348190 |  |  |  |
| *P. sundbergi sundbergi* | 57PL | 102 Valle du Mai, Praslin | KC347830 | KC348191 | KC348312 |  | KC348053 |
| *P. sundbergi sundbergi* | 73PL * | 99 Anse Volbert, Praslin | KC347831 | KC348192 | KC348313 | KC347965 | KC348054 |
| *P. sundbergi sundbergi* | 74PL * | 99 Anse Volbert, Praslin | KC347832 | KC348193 | KC348314 | KC347966 | KC348055 |
| *P. sundbergi sundbergi* | 75PL | 99 Anse Volbert, Praslin | KC347833 | KC348194 |  |  |  |
| *P. sundbergi sundbergi* | 76PL | 99 Anse Volbert, Praslin | KC347834 | KC348195 | KC348316 |  | KC348056 |
| *P. sundbergi sundbergi* | 77PL | 99 Anse Volbert, Praslin | KC347835 | KC348196 |  | KC347967 | KC348057 |
| *P. sundbergi sundbergi* | 78PL | 99 Anse Volbert, Praslin | KC347836 | KC348197 |  | KC347968 |  |
| *P. sundbergi sundbergi* | 79PL * | 99 Anse Volbert, Praslin | KC347837 | KC348198 | KC348317 | KC347969 | KC348058 |
| *P. sundbergi sundbergi* | 80PL * | 99 Anse Volbert, Praslin | KC347838 | KC348199 | KC348315 | KC347970 | KC348059 |
| *P. sundbergi sundbergi* | 13CUR * | 81 Turtle Pond, Curieuse | KC347839 | KC348200 | KC348318 | KC347971 | KC348061 |
|  |  |  |  |  |  |  |  |
| *P. sundbergi ladiguensis* | LD1 * | 93 La Veuve Reserve, La Digue | KC347840 | KC348202 | KC348321 | GU180954 | KC348062 |
| *P. sundbergi ladiguensis* | LD17 * | 95 Belle Vue, La Digue | KC347841 | KC348203 | GU180999 | KC347973 | KC348063 |
| *P. sundbergi ladiguensis* | LD22 * | 94B To Grand Anse, La Digue | KC347842 | KC348204 | KC348320 | KC347974 | KC348064 |
| *P. sundbergi ladiguensis* | LD28 | 93 La Veuve Reserve, La Digue | KC347843 | KC348205 |  |  | KC348065 |
| *P. sundbergi ladiguensis* | 1LD | 93 La Veuve Reserve, La Digue | KC347844 | KC348206 |  |  | KC348066 |
| *P. sundbergi ladiguensis* | 17LD * | 93 La Veuve Reserve, La Digue | KC347845 | KC348207 | KC348319 | KC347972 | KC348067 |
| *P. sundbergi ssp.* | 64LD^a^ | 96 Anse Source d'Argeant, La Digue | KC347846 | KC348208 | KC348322 | KC347975 |  |
| *P. sundbergi ssp.* | 74LD^a^ | 96 Anse Source d'Argeant, La Digue | KC347847 | KC348209 | KC348323 |  | KC348068 |
| *P. sundbergi ladiguensis* | 3BS * | 90 Point 1, Grande Soeur | KC347848 | KC348210 | KC348324 | KC347976 | KC348060 |
|  |  |  |  |  |  |  |  |
| *P. sundbergi longinsulae* | CM5 | 8 Menai Island, Cosmoledo | KC347857 | FJ830319 |  |  |  |
| *P. sundbergi longinsulae* | CM6 | 8 Menai Island, Cosmoledo | KC347858 |  |  |  |  |
| *P. sundbergi longinsulae* | CM7 | 8 Menai Island, Cosmoledo | KC347859 |  |  |  |  |
| *P. sundbergi longinsulae* | CM8 | 8 Menai Island, Cosmoledo | KC347860 |  |  |  |  |
| *P. sundbergi longinsulae* | CM9 | 8 Menai Island, Cosmoledo | KC347861 | KC348183 |  |  |  |
| *P. sundbergi sundbergi* | PV7 | 14 Poivre | KC347862 | KC348201 |  |  |  |
| *P. sundbergi sundbergi* | PV3 | 14 Poivre |  | FJ830317 |  |  |  |
|  |  |  |  |  |  |  |  |
| *P. astriata astriata* | 2MA61 * | 55 Anse aux Forbans, Mahé | KC347867 | KC348211 | KC348325/6^c^ | KC347981 | KC348070 |
| *P. astriata astriata* | 3MA45 * | 59 Baie Lazare, Mahé | KC347868 | KC348212 | GU181001 | GU180955 | KC348069 |
| *P. astriata astriata* | 3MA106 * | 18 NorthEast Point, Mahé | KC347869 | KC348213 | KC348327 | KC347982 | KC348071 |
| *P. astriata astriata* | 3MA158 * | 33 Anse Major, Mahé | KC347870 | KC348214 | KC348328 | KC347983 | KC348072 |
| *P. astriata astriata* | 3MA161 * | 39 Tea Plantation, Mahé | KC347871 | KC348215 | KC348329 | KC347977 | KC348073 |
| *P. astriata astriata* | 3MA170 * | 45 Copolia, Mahé | KC347872 | KC348216 | KC348330 | KC347978 | KC348074 |
| *P. astriata astriata* | 3MA207 * | 53 Anse Louis, Mahé | KC347873 | KC348217 | KC348331 | KC347979 | KC348075 |
| *P. astriata astriata* | 6MA * | 29 Mare aux Cochons 2, Mahé | KC347874 | KC348218 | KC348332 | KC347980 | KC348076 |
| *P. astriata astriata* | CF7 * | 70 Cerf Island 2 | KC347875 | KC348219 | KC348333/4^c^ | KC347984 | KC348077 |
| *P. astriata astriata* | 38710A * | 71 La Passe 1, Silhouette | KC347876 | KC348220 | KC348335 | KC347985 | KC348078 |
| *P. astriata astriata* | 38712 | 71 La Passe 1, Silhouette | KC347877 | KC348221 |  | KC347986 |  |
| *P. astriata astriata* | PHELSUN1 | 75 Anse Lascars, Silhouette | KC347878 | KC348222 |  | KC347987 |  |
| *P. astriata astriata* | PHELSUN2 | 75 Anse Lascars, Silhouette | KC347879 | KC348223 |  |  |  |
| *P. astriata astriata* | PHELSUN3 | 75 Anse Lascars, Silhouette | KC347880 | KC348224 |  |  |  |
| *P. astriata astriata* | 38711 | 75 Anse Lascars, Silhouette | KC347881 | KC348225 |  |  |  |
| *P. astriata astriata* | 8SILH * | 75 Anse Lascars, Silhouette | KC347882 | KC348226 | GU181002 |  | KC348079 |
| *P. astriata astriata* | 9SILH * | 75 Anse Lascars, Silhouette | KC347883 | KC348227 | KC348336/7^c^ |  | KC348080 |
| *P. astriata astriata* | 10SILH * | 75 Anse Lascars, Silhouette | KC347884 | KC348228 | KC348338 | GU180956 | KC348081 |
| *P. astriata astriata* | 14SILH * | 75 Anse Lascars, Silhouette | KC347885 | KC348229 | KC348339/40^c^ | KC347988 | KC348082 |
| *P. astriata astriata* | 15SILH * | 75 Anse Lascars, Silhouette | KC347886 | KC348230 | KC348341/2^c^ | KC347989 | KC348083 |
|  |  |  |  |  |  |  |  |
| *P. astriata semicarinata* | PL6 | 99 Anse Volbert, Praslin | KC347887 | KC348234 |  |  |  |
| *P. astriata semicarinata* | PL9 * | 99 Anse Volbert, Praslin | KC347888 | KC348235 |  | GU180958 | KC348087 |
| *P. astriata semicarinata* | PL15 * | 103 Anse Volbert 3, Praslin | KC347889 | KC348236 |  | KC347992 | KC348088 |
| *P. astriata semicarinata* | PL26 | 102 Valle du Mai, Praslin | KC347890 | KC348237 |  |  |  |
| *P. astriata semicarinata* | PL27 | 107 Anse Citron, Praslin | KC347891 | KC348238 |  | KC347993 | KC348089 |
| *P. astriata semicarinata* | PL30 | 110 Fonde de L'Anse, Praslin | KC347892 | KC348239 |  | KC347994 |  |
| *P. astriata semicarinata* | PL34 | 99 Anse Volbert, Praslin | KC347893 | KC348240 |  |  |  |
| *P. astriata semicarinata* | PHEASTPRA1 | 109 Grand Anse, Praslin | KC347894 | KC348241 |  | KC347995 |  |
| *P. astriata semicarinata* | 2PL * | 109 Grand Anse, Praslin | KC347895 | KC348242 | GU181003 | KC347996 | KC348090 |
| *P. astriata semicarinata* | 24PL | 99 Anse Volbert, Praslin | KC347896 | KC348243 | KC348344 |  |  |
| *P. astriata semicarinata* | 25PL | 105 Mont Plaisir, Praslin | KC347897 | KC348244 | KC348345 |  |  |
| *P. astriata semicarinata* | 26PL | 105 Mont Plaisir, Praslin | KC347898 | KC348245 |  |  |  |
| *P. astriata semicarinata* | 27PL | 105 Mont Plaisir, Praslin | KC347899 | KC348246 |  | KC347998 |  |
| *P. astriata semicarinata* | 28PL * | 105 Mont Plaisir, Praslin | KC347900 | KC348247 | KC348343 | KC347997 | KC348086 |
| *P. astriata semicarinata* | 37PL | 110 Fonde de L'Anse, Praslin | KC347901 | KC348248 |  |  |  |
| *P. astriata semicarinata* | 45PL * | 113 Anse Marie Louise, Praslin | KC347902 | KC348249 | KC348346 | KC347999 | KC348091 |
| *P. astriata semicarinata* | 65PL | 102 Valle du Mai, Praslin | KC347903 | KC348250 | KC348347 |  |  |
| *P. astriata semicarinata* | 66PL * | 102 Valle du Mai, Praslin | KC347904 | KC348251 | KC348348 | KC348000 | KC348092 |
| *P. astriata semicarinata* | 68PL * | 100 Anse Volbert 2, Praslin | KC347905 | KC348252 | KC348349 | KC348001 | KC348093 |
| *P. astriata semicarinata* | 69PL | 100 Anse Volbert 2, Praslin | KC347906 | KC348253 |  |  |  |
| *P. astriata semicarinata* | 70PL | 100 Anse Volbert 2, Praslin | KC347907 | KC348254 | KC348350 | KC348002 |  |
| *P. astriata semicarinata* | 71PL | 100 Anse Volbert 2, Praslin | KC347908 | KC348255 | KC348351 |  |  |
| *P. astriata semicarinata* | 72PL | 100 Anse Volbert 2, Praslin | KC347909 | KC348256 |  | KC348003 | KC348094 |
| *P. astriata semicarinata* | 81PL * | 104 Anse La Blague, Praslin | KC347910 | KC348257 | KC348352 | KC348004 | KC348095 |
| *P. astriata semicarinata* | LD14 * | 95 Belle Vue, La Digue | KC347911 | KC348258 | KC348365 | KC348017 | KC348107 |
| *P. astriata semicarinata* | LD19 | 95 Belle Vue, La Digue | KC347912 | KC348259 | KC348366 |  | KC348108 |
| *P. astriata semicarinata* | LD20 | 94 La Veuve Reserve 2, La Digue | KC347913 | KC348260 |  | KC348018 | KC348109 |
| *P. astriata semicarinata* | 3LD * | 93 La Veuve Reserve, La Digue | KC347914 | KC348261 | KC348363 | KC348015 | KC348110 |
| *P. astriata semicarinata* | 15LD * | 93 La Veuve Reserve, La Digue | KC347915 | KC348262 | KC348364 | KC348016 | KC348111 |
| *P. astriata semicarinata* | CUR2 | 82 Trail, Curieuse | KC347916 | KC348263 |  |  |  |
| *P. astriata semicarinata* | CUR6 | 81 Turtle Pond, Curieuse | KC347917 | KC348264 |  |  |  |
| *P. astriata semicarinata* | 3CUR * | 80 Point1, Curieuse | KC347918 | KC348265 | KC348353 | KC348009 | KC348102 |
| *P. astriata semicarinata* | 6CUR | 80 Point1, Curieuse | KC347919 | KC348266 | KC348354 | KC348010 |  |
| *P. astriata semicarinata* | 7CUR | 81 Turtle Pond, Curieuse | KC347920 | KC348267 |  | KC348011 | KC348103 |
| *P. astriata semicarinata* | 8CUR * | 81 Turtle Pond, Curieuse | KC347921 | KC348268 | KC348355 | KC348012 | KC348104 |
| *P. astriata semicarinata* | 15CUR * | 80 Point1, Curieuse | KC347922 | KC348269 | KC348356 | KC348013 | KC348105 |
| *P. astriata semicarinata* | 40CUR * | 80 Point1, Curieuse | KC347923 | KC348270 | KC348357 | KC348014 | KC348106 |
| *P. astriata semicarinata* | 17ARD * | 79 Aride Island | KC347924 | KC348277 | GU181004 | KC348019 | KC348112 |
| *P. astriata semicarinata* | 22ARD | 79 Aride Island | KC347925 | KC348278 |  | KC348020 | KC348113 |
| *P. astriata semicarinata* | 7CNE * | 89 To Cave, Cousine | KC347926 | KC348271 | GU181005 | GU180959 | KC348097 |
| *P. astriata semicarinata* | 8CNE * | 89 To Cave, Cousine | KC347927 | KC348272 | KC348358 | KC348005 | KC348096 |
| *P. astriata semicarinata* | 40CNE * | 87 Office, Cousine | KC347928 | KC348273 | KC348359 | KC348006 | KC348098 |
| *P. astriata semicarinata* | 41CNE | 87 Office, Cousine | KC347929 | KC348274 | KC348360 |  | KC348099 |
| *P. astriata semicarinata* | 42CNE * | 87 Office, Cousine | KC347930 | KC348275 | KC348361 | KC348007 | KC348100 |
| *P. astriata semicarinata* | 43CNE * | 87 Office, Cousine | KC347931 | KC348276 | KC348362 | KC348008 | KC348101 |
| *P. astriata semicarinata* | 11BS * | 92 north path 2, Grande Soeur | KC347932 | KC348279 | KC348367 | KC348021 | KC348114 |
|  |  |  |  |  |  |  |  |
| *P. astriata ssp.* | 23FG * | 114 Fregate | KC347933 | KC348231 | KC348369 | KC347991 | KC348084 |
| *P. astriata ssp.* | 10FG * | 114 Fregate | KC347934 | KC348232 | KC348370 | KC347990 | KC348085 |
| *P. astriata ssp.* | 16FG | 114 Fregate | KC347935 | KC348233 | KC348368 | GU180957 |  |
|  |  |  |  |  |  |  |  |
| *P. astriata astriata* | AT6 | 7 Astove | KC347863 | FJ830248 |  |  |  |
| *P. astriata astriata* | AT15 | 7 Astove | KC347864 | FJ830249 |  |  |  |
| *P. astriata astriata* | APH1^b^ | 13 Alphonse | KC347865 | FJ830250 |  |  |  |
| *P. astriata astriata* | APH10^b^ | 13 Alphonse | KC347866 | FJ830251 |  |  |  |

Reference:

Rocha S., Harris, D.J., Perera, A., Silva, A., Vasconcelos, R., Carretero, M. A. (2009) Recent data on the distribution of Lizards and Snakes of the Seychelles. *Herpetological Bulletin*, **110**, 20-32.

^a^ Note that for individuals 3MA206; 64LD and 74LD (P. sundbergi) the subspecies is not assigned has they clustered with an unexpected subspecies at mtDNA (see results and discussion at manuscript)

^b^ Note that subspecific status corrects the previously reported in Rocha et al. (2009) for this same individuals.

^c^ The two accession numbers of some individuals for the marker *Relnint61*, correspond to individual haplotypes read directly from sequence data
